# Regional Changes in Charcoal-Burning Suicide Rates in East/Southeast Asia from 1995 to 2011: A Time Trend Analysis

**DOI:** 10.1371/journal.pmed.1001622

**Published:** 2014-04-01

**Authors:** Shu-Sen Chang, Ying-Yeh Chen, Paul S. F. Yip, Won Jin Lee, Akihito Hagihara, David Gunnell

**Affiliations:** 1 Hong Kong Jockey Club Centre for Suicide Research and Prevention, The University of Hong Kong, Pokfulam, Hong Kong SAR, China; 2 Department of Social Work and Social Administration, The University of Hong Kong, Pokfulam, Hong Kong SAR, China; 3 Ju Shan Hospital, Taoyuan, Taiwan; 4 School of Social and Community Medicine, University of Bristol, Bristol, United Kingdom; 5 Taipei City Psychiatric Center, Taipei City Hospital, Taipei City, Taiwan; 6 Institute of Public Health and Department of Public Health, National Yang-Ming University, Taipei City, Taiwan; 7 Department of Preventive Medicine, College of Medicine, Korea University, Seoul, Republic of Korea; 8 Department of Health Services Management and Policy, Kyushu University Graduate School of Medicine, Fukuoka, Japan; University of Western Sydney, Australia

## Abstract

**Background:**

Suicides by carbon monoxide poisoning resulting from burning barbecue charcoal reached epidemic levels in Hong Kong and Taiwan within 5 y of the first reported cases in the early 2000s. The objectives of this analysis were to investigate (i) time trends and regional patterns of charcoal-burning suicide throughout East/Southeast Asia during the time period 1995–2011 and (ii) whether any rises in use of this method were associated with increases in overall suicide rates. Sex- and age-specific trends over time were also examined to identify the demographic groups showing the greatest increases in charcoal-burning suicide rates across different countries.

**Methods and Findings:**

We used data on suicides by gases other than domestic gas for Hong Kong, Japan, the Republic of Korea, Taiwan, and Singapore in the years 1995/1996–2011. Similar data for Malaysia, the Philippines, and Thailand were also extracted but were incomplete. Graphical and joinpoint regression analyses were used to examine time trends in suicide, and negative binomial regression analysis to study sex- and age-specific patterns. In 1995/1996, charcoal-burning suicides accounted for <1% of all suicides in all study countries, except in Japan (5%), but they increased to account for 13%, 24%, 10%, 7%, and 5% of all suicides in Hong Kong, Taiwan, Japan, the Republic of Korea, and Singapore, respectively, in 2011. Rises were first seen in Hong Kong after 1998 (95% CI 1997–1999), followed by Singapore in 1999 (95% CI 1998–2001), Taiwan in 2000 (95% CI 1999–2001), Japan in 2002 (95% CI 1999–2003), and the Republic of Korea in 2007 (95% CI 2006–2008). No marked increases were seen in Malaysia, the Philippines, or Thailand. There was some evidence that charcoal-burning suicides were associated with an increase in overall suicide rates in Hong Kong, Taiwan, and Japan (for females), but not in Japan (for males), the Republic of Korea, and Singapore. Rates of change in charcoal-burning suicide rate did not differ by sex/age group in Taiwan and Hong Kong but appeared to be greatest in people aged 15–24 y in Japan and people aged 25–64 y in the Republic of Korea. The lack of specific codes for charcoal-burning suicide in the International Classification of Diseases and variations in coding practice in different countries are potential limitations of this study.

**Conclusions:**

Charcoal-burning suicides increased markedly in some East/Southeast Asian countries (Hong Kong, Taiwan, Japan, the Republic of Korea, and Singapore) in the first decade of the 21st century, but such rises were not experienced by all countries in the region. In countries with a rise in charcoal-burning suicide rates, the timing, scale, and sex/age pattern of increases varied by country. Factors underlying these variations require further investigation, but may include differences in culture or in media portrayals of the method.

*Please see later in the article for the Editors' Summary*

## Abstract 2

Using a time trend analysis, Ying-Yeh Chen and colleagues examine the evidence for regional increases in charcoal-burning suicide rates in East and Southeast Asia from 1995 to 2011.

*Please see later in the article for the Editors' Summary*

## Editors' Summary

**Background:**

Every year, almost one million people die by suicide globally; suicide is the fifth leading cause of death in women aged 15–49 and the sixth leading cause of death in men in the same age group. Most people who take their own life are mentally ill. For others, stressful events (the loss of a partner, for example) have made life seem worthless or too painful to bear. Strategies to reduce suicide rates include better treatment of mental illness and programs that help people at high risk of suicide deal with stress. Suicide rates can also be reduced by limiting access to common suicide methods. These methods vary from place to place. Hanging is the predominant suicide method in many countries, but in Hong Kong, for example, jumping from a high building is the most common method. Suicide methods also vary over time. For example, after a woman in Hong Kong took her life in 1998 by burning barbecue charcoal in a sealed room (a process that produces the toxic gas carbon monoxide), charcoal burning rapidly went from being a rare method of killing oneself in Hong Kong to the second most common suicide method.

**Why Was This Study Done?:**

Cases of charcoal-burning suicide have also been reported in several East and Southeast Asian countries, but there has been no systematic investigation of time trends and regional patterns of this form of suicide. A better understanding of regional changes in the number of charcoal-burning suicides might help to inform efforts to prevent the emergence of other new suicide methods. Here, the researchers investigate the time trends and regional patterns of charcoal-burning suicide in several countries in East and Southeast Asia between 1995 and 2011 and ask whether any rises in the use of this method are associated with increases in overall suicide rates. The researchers also investigate sex- and age-specific time trends in charcoal-burning suicides to identify which groups of people show the greatest increases in this form of suicide across different countries.

**What Did the Researchers Do and Find?:**

The researchers analyzed method-specific data on suicide deaths for Hong Kong, Japan, the Republic of Korea, Taiwan, and Singapore between 1995/1996 and 2011 obtained from the World Health Organization Mortality Database and from national death registers. In 1995/1996, charcoal-burning suicides accounted for less than 1% of all suicides in all these countries except Japan (4.9%). By 2011, charcoal-burning suicides accounted for between 5% (Singapore) and 24% (Taiwan) of all suicides. Rises in the rate of charcoal-burning suicide were first seen in Hong Kong in 1999, in Singapore in 2000, in Taiwan in 2001, in Japan in 2003, and in the Republic of Korea in 2008. By contrast, incomplete data from Malaysia, the Philippines, and Thailand showed no evidence of a marked increase in charcoal-burning suicide in these countries over the same period. Charcoal-burning suicides were associated with an increase in overall suicide rates in Hong Kong in 1998–2003, in Taiwan in 2000–2006, and in Japanese women after 2003. Finally, the annual rate of change in charcoal-burning suicide rate did not differ by sex/age group in Taiwan and Hong Kong, whereas in Japan people aged 15–24 and in the Republic of Korea people aged 25–64 tended to have the greatest rates of increase.

**What Do These Findings Mean?:**

These findings show that charcoal-burning suicides increased markedly in several but not all East and Southeast Asian countries during the first decade of the 21st century. Moreover, in countries where there was an increase, the timing, scale, and sex/age pattern of the increase varied by country. The accuracy of these findings is likely to be limited by several aspects of the study. For example, because of the way that method-specific suicides are recorded in the World Health Organization Mortality Database and national death registries, the researchers may have slightly overestimated the number of charcoal-burning suicides. Further studies are now needed to identify the factors that underlie the variations between countries in charcoal-burning suicide rates and time trends reported here. However, the current findings highlight the need to undertake surveillance to identify the emergence of new suicide methods and the importance of policy makers, the media, and internet service providers working together to restrict graphic and detailed descriptions of new suicide methods.

**Additional Information:**

Please access these websites via the online version of this summary at http://dx.doi.org/10.1371/journal.pmed.1001622.

- A *PLOS Medicine* research article by Shu-Sen Chang and colleagues investigates time trends and regional patterns of charcoal-burning suicide in Taiwan
- The World Health Organization provides information on the global burden of suicide and on suicide prevention (in several languages); it also has an article on international patterns in methods of suicide
- The US National Institute of Mental Health provides information on suicide and suicide prevention
- The UK National Health Service Choices website has detailed information about suicide and its prevention
- MedlinePlus provides links to further resources about suicide (in English and Spanish)
- The International Association for Suicide Prevention provides links to crisis centers in Asia
- The charity Healthtalkonline has personal stories about dealing with suicide

## Introduction

Globally, suicide is amongst the leading causes of premature mortality; in 2010, it was the fifth leading cause of death in women and the sixth in men among individuals aged 15–49 y [1]. Time trends in suicide rates may be influenced by a number of factors including socio-economic changes, the prevalence of mental illness or distress, and certain types of media reporting [2]; there is growing evidence that changes in the popularity and availability of lethal suicide methods could also have a marked impact on time trends in overall suicide rates [3]–[6].

Previous studies of method availability and suicide have mostly focused on the impact of restricting access to methods [5], such as detoxification of domestic gas [6], bans on sales of toxic pesticides [3],[7], and legal changes in firearms regulations [8],[9]. However, many suicides using these methods had already occurred before the implementation of restrictions, highlighting the potential importance of surveillance for the emergence of new suicide methods at an early stage to enable public health action to prevent an increase of suicide by new methods [4],[5].

In 1998–2000 there was a rapid rise in suicide by carbon monoxide poisoning from the inhalation of barbecue charcoal gas in Hong Kong and Taiwan [10]–[12]. Suicides by this method used to be very rare, but within 5 y charcoal burning became the second most common method of suicide in these two countries. Although cases of charcoal-burning suicide have been reported in other neighbouring East/Southeast Asian countries such as China [13], Japan [14], Macao [15], Malaysia [16], Singapore [17], and the Republic of Korea [18], to the best of our knowledge, there has been no systematic investigation of regional patterns and time trends in the use of this method and the association between time trends in charcoal-burning suicide and overall suicide rates in affected countries.

We used data from eight East/Southeast Asian countries (Hong Kong, Taiwan, Japan, the Republic of Korea, Singapore, Malaysia, the Philippines, and Thailand) to investigate time trends in charcoal-burning suicide across different countries and the association between changes in charcoal-burning suicide and overall suicide rates for the years 1995–2011. We also examined sex- and age-specific time trends to identify the demographic groups showing the greatest increases in charcoal-burning suicide rates across different countries. Our overall aim was to establish what can be learnt from the changing incidence of charcoal-burning suicide in this region to inform the prevention of the future emergence of novel suicide methods. Specifically, the objectives of this analysis were to investigate (i) time trends and regional patterns of charcoal-burning suicide throughout East/Southeast Asia during the period 1995–2011 and (ii) whether any rises in use of this method were associated with increases in overall suicide rates. Sex- and age-specific trends over time were also examined to identify the demographic groups showing the greatest increases in charcoal-burning suicide rates across different countries.

## Methods

### Ethics Statement

The study used only aggregate secondary data that were available openly; no identifiable personal data were used in the study. Ethical approval was thus not required.

### Data

**Figure 1:**
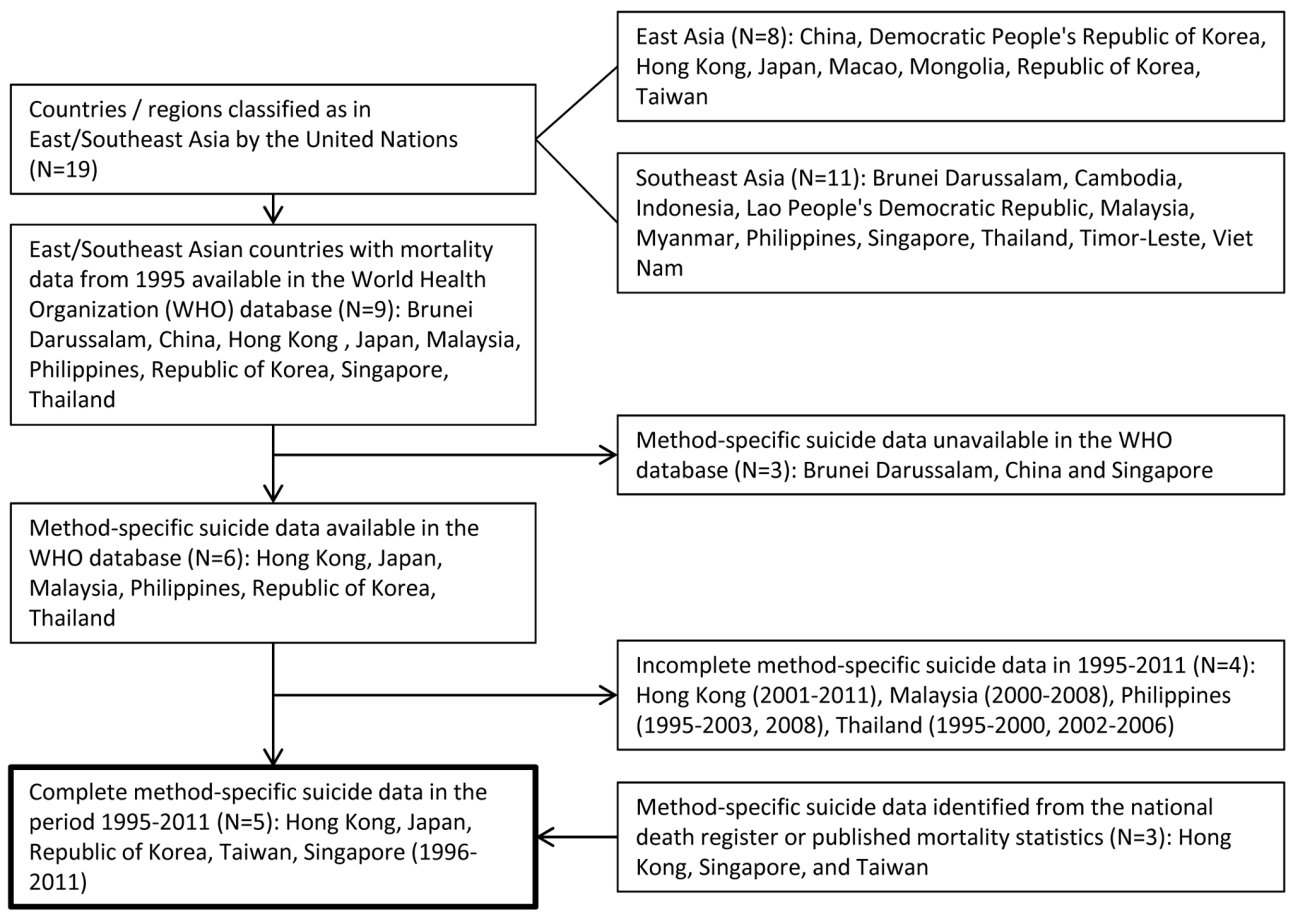
Flow chart of how data were identified for study countries in East/Southeast Asia.

To investigate time trends in charcoal-burning suicide in East/Southeast Asia we first systematically identified countries with data available in the World Health Organization (WHO) Mortality Database [19], which provides the most comprehensive standardised national mortality statistics for countries around the world. Figure 1 shows a flow chart summarising how we identified data for the study countries. In brief, we first identified 19 countries that were classified as in the East/Southeast Asia region by the United Nations (eight in East Asia and 11 in Southeast Asia) [20]. The WHO Mortality Database contained suicide data for nine of these countries, but only six had method-specific data available. We then extracted complete method-specific suicide data by sex, age (5-y bands), and year for Japan and the Republic of Korea for the period 1995–2011, and for the years available for Hong Kong (2001–2011), Malaysia (2000–2008), the Philippines (1995–2003, 2008), and Thailand (1995–2000, 2002–2006). Data for the 3-y period (1995–1997) prior to 1998, when the first widely publicised suicide by charcoal burning occurred [21],[22], were used to assess the baseline rates. We then supplemented the WHO data by extracting relevant suicide data from the national death registers for Hong Kong (1995–2011) and Taiwan (1995–2011), as well as from published mortality statistics for Singapore (1996–2011), although only sex- and method-specific, but not age-specific, data were available for Singapore [23]. The main analysis therefore included five countries (Hong Kong, Taiwan, Japan, the Republic of Korea, and Singapore) with complete data for the years 1995/1996–2011; suicide data for these countries are thought to be relatively reliable [24]. In contrast, the quality of suicide data for countries with incomplete time series (Malaysia, the Philippines, and Thailand) is thought to be relatively poor [25]–[27]. The WHO Mortality Database also provided population data; when these were incomplete or unavailable, relevant data were extracted from the United Nations population database [28].

There is no specific code for charcoal-burning suicide in the International Classification of Diseases (ICD) [29]. We used the following ICD codes to extract data for charcoal-burning suicide: E952 in the ninth revision of ICD (ICD-9) and X67 in the tenth revision of ICD (ICD-10). These codes include suicides by poisoning using gases other than domestic gas and are not restricted to charcoal-burning suicides. Previous studies showed that more than 90% of deaths in this category were charcoal-burning suicides in Hong Kong [10] and Taiwan [30]; these studies used data from 2002 or before, but results were similar when we examined data for 2010, the most recent year for which detailed cause-of-death information was available—99% and 85% of suicides coded as ICD-10 X67 were charcoal-burning suicides in Hong Kong and Taiwan, respectively. We did not have access to data from other countries to investigate this further. For simplicity, we refer to ICD-9 E952 and ICD-10 X67 deaths as charcoal-burning suicides throughout the paper, although not all cases used this method. For example, in Japan some of deaths with these ICD codes were due to hydrogen sulphide poisoning when the use of this method rapidly increased in 2008 [31]. Suicides by all other methods were identified using the following codes, excluding charcoal-burning suicides as defined above: ICD-9 E950–E959 and ICD-10 X60–X84. Previous studies have indicated that many deaths categorised as undetermined intent are likely to be misclassified suicides [32]–[35], including charcoal-burning suicides [30]; therefore, we included undetermined deaths in the main analysis. The following codes were used to identify such deaths: (i) ICD-9 E982 and ICD-10 Y17 for deaths by charcoal burning and (ii) ICD-9 E980–E989 and ICD-10 Y10–Y34, excluding the previously defined codes, for suicides by other methods. Accidents by non-domestic gas (carbon monoxide included) were excluded, as one analysis showed that they had different demographic characteristics from those of suicides, suggesting that they were not misclassified suicides [32]. In sensitivity analyses we used data for certified suicides only (Tables S1 and S2).

### Statistical Analysis

Age-standardised suicide rates for people aged 15 y or above were calculated using the WHO world standard population [36], when age-specific data were available. We used joinpoint regression (a segmented line regression or piecewise linear regression) to investigate time trends in charcoal-burning suicide and their associations with changes in overall suicide rates; this approach has been widely used to study time trends in mortality from causes such as cancer [37] and suicide [4],[38]–[44]. Analyses were carried out using the the Joinpoint Regression Program (version 4.0.4, US National Cancer Institute) [45]; this software has been used by the US National Cancer Institute in the analyses of cancer trends published in their regular reports. In joinpoint regression analysis, suicide time trends are characterised by contiguous linear segments and “join points” (points at which trends change). The grid search method proposed by Lerman [46] was used to fit the segmented line regression; this method creates a “grid” of all possible locations for join points and tests the sum of squared errors for each one to find the best possible fit. This method allows the join points to occur only exactly at observed time points, not between time points. To identify the combination of join points and linear segments that fits the time series data best, a sequence of Monte Carlo permutation tests was used when comparing pairs of models differing by one join point [47]. The sequence started by comparing the model with zero join points (i.e., a straight line with no change in trend) and that with one join point, and it ended when there was no statistical evidence that more joint points fit the data better or when reaching the maximum number of join points allowed. The number of join points was limited to a maximum of three according to the Joinpoint Regression Program guidelines based on the number of data points in the datasets [45]. The overall probability of type 1 error (i.e., concluding that there are one or more join points when there are in fact none) was maintained at 0.05. For the majority of models the age-standardised rate was used as the dependent variable, and thus the regression coefficient indicated the mean increase in rate per year within segments identified by joinpoint regression analysis. In Hong Kong, there were zero values for charcoal-burning suicide in some years, causing some problems in estimating the standard errors of age-standardised rates; therefore, crude rates with variance based on the Poisson distribution were modelled, with the suicide counts adjusted by the addition of a small constant, i.e., 0.5 [45]. Crude rates for Singapore were modelled similarly, as age-specific suicide data were unavailable. In a sensitivity analysis the autocorrelation was accounted for by estimating the regression coefficients using weighted least squares in all models [47].

Negative binomial regression models were used to estimate the annual rate of change in charcoal-burning suicide rates by sex for all ages and four age groups (15–24, 25–44, 45–64, and 65+ y), for the time periods (i) when there were rises in rates, i.e., from the start of the rise to its peak or to the most recent year when data were available, and (ii) when there were reductions in rates, i.e., from the peak to the most recent year when data were available. The starting and peak years were identified in the joinpoint regression analyses and by visual inspection of the graphs of time trends in charcoal-burning suicide. Negative binomial regression models were used because there was evidence for over-dispersion in the Poisson regression analyses of the data. We calculated incidence rate ratios assuming a linear change in rates. To examine whether the annual rate of change in charcoal-burning suicide differed between sexes or amongst people of different age groups, interaction terms between sex and year or between age group and year were included in the models. Negative binomial regression models were estimated using Stata version 12 (StataCorp).

## Results

**Figure 2:**
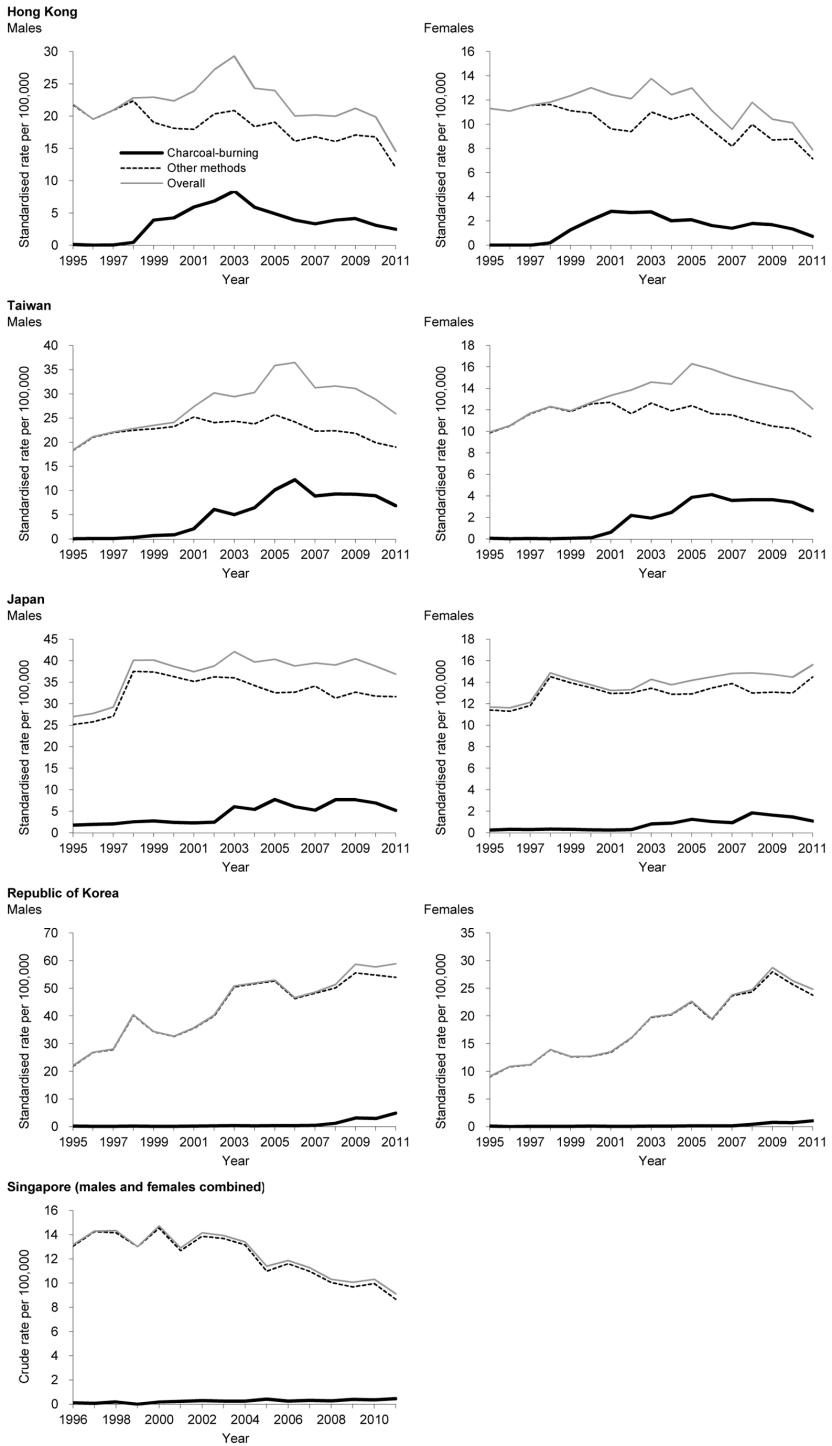
Time trends in suicide rates: overall suicide, charcoal-burning suicide, and suicide by other methods.

Figure 2 shows time trends in overall suicide, charcoal-burning suicide, and suicide by all other methods in five East/Southeast Asian countries with complete data for 1995/1996–2011. Charcoal-burning suicide rates increased in all countries over the study period, but the magnitude of the rise varied by country. Rates were generally very low in 1995/1996, ranging from 0.1 to 1.1 per 100,000 (Table 1), whilst in 2011 they increased to values ranging from 0.4 per 100,000 (Singapore) to 4.8 per 100,000 (Taiwan). In 1995/1996, charcoal-burning suicides accounted for only 0.4%, 0.6%, 4.9%, 0.7%, and 0.8% of all suicides in Hong Kong, Taiwan, Japan, the Republic of Korea, and Singapore, respectively. These figures rose to 13.4%, 24.1%, 9.7%, 7.0%, and 4.9%, respectively, in 2011. The magnitude of increase over the period 1995/1996–2011 was most marked in Taiwan (absolute increase in percentage, 23.5%; 65-fold increase in rate) and Hong Kong (13.0%; 27-fold), followed by the Republic of Korea (6.2%; 28-fold), Japan (4.8%; 3-fold), and Singapore (4.0%; 4-fold). However, it should be noted that these estimates are sensitive to baseline rates. Rises in charcoal-burning suicide were seen in both males and females (Figure 2; Table 1). The patterns for data based only on certified suicides were similar (Figure S1; Table S1). In Malaysia, the Philippines, and Thailand, data were less complete than in the five countries reported above (Table 2). Although there was no indication of a marked rise in charcoal-burning suicide rate in these three countries, a very slight rise in Malaysia was observed (the rate increased from 0.06 per 100,000 in 2000 to a peak of 0.30 per 100,000 in 2003). For the most recent year with data available, the rate per 100,000 was 0.13 in Malaysia (2008), 0.01 in the Philippines (2008), and 0.00 in Thailand (2006).

**Table 1:** Number, percent, and rate per 100,000 of charcoal-burning suicide (coded as ICD-9 E952 or E982, or ICD-10 X67 or Y17).

Year	Hong Kong			Taiwan			Japan^a^			Republic of Korea			Singapore		
	Number	Percent	Rate	Number	Percent	Rate	Number	Percent	Rate	Number	Percent	Rate	Number	Percent	Rate
**Males and females**															
1995	3	0.4%	0.1	13	0.6%	0.1	1,091	4.9%	1.0	38	0.7%	0.1			
1996	0	0.0%	0.0	12	0.5%	0.1	1,213	5.2%	1.1	20	0.3%	0.1	4	0.8%	0.1
1997	1	0.1%	0.0	15	0.6%	0.1	1,281	5.2%	1.2	30	0.5%	0.1	2	0.4%	0.1
1998	21	2.2%	0.3	32	1.1%	0.2	1,618	4.9%	1.4	32	0.3%	0.1	7	1.2%	0.2
1999	149	15.1%	2.6	68	2.3%	0.4	1,691	5.2%	1.5	26	0.3%	0.1	0	0.0%	0.0
2000	179	17.9%	3.1	88	2.8%	0.5	1,482	4.7%	1.3	28	0.4%	0.1	7	1.2%	0.2
2001	257	24.2%	4.3	259	7.2%	1.4	1,414	4.6%	1.2	43	0.5%	0.1	9	1.7%	0.2
2002	277	24.4%	4.7	773	19.5%	4.2	1,556	4.9%	1.4	68	0.7%	0.2	12	2.0%	0.3
2003	325	25.6%	5.4	660	16.4%	3.5	3,662	10.8%	3.4	85	0.7%	0.2	10	1.7%	0.2
2004	228	20.7%	3.8	847	20.2%	4.5	3,300	10.3%	3.1	70	0.5%	0.2	10	1.8%	0.2
2005	217	18.9%	3.4	1,338	27.0%	7.0	4,603	14.2%	4.4	92	0.6%	0.2	17	3.5%	0.4
2006	165	16.9%	2.7	1,576	31.2%	8.2	3,575	11.2%	3.5	97	0.8%	0.2	11	2.1%	0.2
2007	143	15.3%	2.3	1,213	26.6%	6.3	3,131	9.5%	3.1	115	0.8%	0.3	14	2.7%	0.3
2008	167	16.5%	2.8	1,268	27.5%	6.5	4,432	13.8%	4.7	336	2.2%	0.8	13	2.6%	0.3
2009	181	17.7%	2.8	1,271	27.7%	6.4	4,454	13.6%	4.6	834	4.5%	2.0	19	3.8%	0.4
2010	138	13.9%	2.2	1,232	27.9%	6.2	4,042	12.7%	4.1	782	4.3%	1.8	18	3.4%	0.4
2011	101	13.4%	1.5	957	24.1%	4.8	2,993	9.7%	3.1	1,290	7.0%	3.0	23	4.9%	0.4
**Males**															
1995	3	0.6%	0.1	7	0.5%	0.1	961	6.5%	1.8	25	0.7%	0.1			
1996	0	0.0%	0.0	10	0.6%	0.1	1,051	6.8%	1.9	17	0.4%	0.1			
1997	1	0.2%	0.0	11	0.6%	0.1	1,114	6.7%	2.0	23	0.5%	0.1			
1998	14	2.3%	0.5	29	1.5%	0.3	1,432	6.2%	2.6	26	0.4%	0.1			
1999	111	17.8%	3.9	62	3.1%	0.7	1,506	6.5%	2.7	17	0.3%	0.1			
2000	116	19.0%	4.2	79	3.8%	0.9	1,332	5.9%	2.3	15	0.3%	0.1			
2001	167	24.9%	5.9	200	8.2%	2.1	1,269	5.8%	2.2	32	0.5%	0.2			
2002	195	25.7%	6.8	572	20.9%	6.1	1,389	6.1%	2.4	58	0.8%	0.3			
2003	238	28.4%	8.4	479	17.6%	5.1	3,234	13.2%	5.9	67	0.8%	0.4			
2004	165	23.7%	5.9	615	21.6%	6.5	2,861	12.3%	5.3	54	0.6%	0.3			
2005	146	20.5%	4.9	973	28.4%	10.2	3,990	17.0%	7.5	66	0.7%	0.3			
2006	112	18.7%	3.9	1,182	33.4%	12.3	3,062	13.6%	5.9	71	0.8%	0.4			
2007	96	15.9%	3.3	871	28.3%	8.9	2,684	11.6%	5.2	83	0.9%	0.4			
2008	108	18.1%	3.9	914	28.9%	9.3	3,649	16.1%	7.5	259	2.6%	1.2			
2009	120	18.6%	4.1	922	29.2%	9.2	3,721	15.9%	7.5	678	5.7%	3.1			
2010	92	15.0%	3.1	896	30.1%	9.0	3,377	15.0%	6.8	642	5.3%	2.9			
2011	76	16.6%	2.5	695	25.8%	6.9	2,496	11.7%	5.1	1,072	8.5%	5.0			
**Females**															
1995	0	0.0%	0.0	6	0.8%	0.1	130	1.7%	0.2	13	0.8%	0.1			
1996	0	0.0%	0.0	2	0.2%	0.0	162	2.1%	0.3	3	0.2%	0.0			
1997	0	0.0%	0.0	4	0.4%	0.0	167	2.1%	0.3	7	0.3%	0.0			
1998	7	2.0%	0.2	3	0.3%	0.0	186	1.9%	0.3	6	0.2%	0.0			
1999	38	10.6%	1.3	6	0.6%	0.1	185	2.0%	0.3	9	0.4%	0.0			
2000	63	16.2%	2.1	9	0.8%	0.1	150	1.6%	0.3	13	0.5%	0.1			
2001	90	23.1%	2.8	59	5.1%	0.6	145	1.7%	0.3	11	0.4%	0.1			
2002	82	21.9%	2.7	201	16.4%	2.2	167	1.9%	0.3	10	0.3%	0.1			
2003	87	20.1%	2.8	181	13.8%	2.0	428	4.6%	0.8	18	0.5%	0.1			
2004	63	15.5%	2.0	232	17.4%	2.5	439	4.9%	0.8	16	0.4%	0.1			
2005	71	16.1%	2.1	365	23.8%	3.9	613	6.8%	1.2	26	0.6%	0.1			
2006	53	14.1%	1.6	394	26.0%	4.1	513	5.6%	1.0	26	0.6%	0.1			
2007	47	14.2%	1.4	342	23.2%	3.6	447	4.7%	0.9	32	0.6%	0.1			
2008	59	14.3%	1.8	354	24.3%	3.7	783	8.4%	1.8	77	1.4%	0.4			
2009	61	16.0%	1.7	349	24.4%	3.7	733	7.9%	1.6	156	2.4%	0.8			
2010	46	12.2%	1.4	336	23.4%	3.4	665	7.1%	1.4	140	2.3%	0.7			
2011	25	8.5%	0.7	262	20.4%	2.7	497	5.1%	1.1	218	3.7%	1.1			

Rate per 100,000 is age-standardised, except Singapore, for which rates were crude rates.

a In Japan, figures for 2008 onwards included not only charcoal-burning suicides but also some deaths from hydrogen sulphide poisoning, which increased markedly in 2008 [31].

**Table 2:** Number, percent, and age-standardised rate per 100,000 of charcoal-burning suicide (coded as ICD-10 X67 or Y17) in Malaysia, the Philippines, and Thailand.

Year	Malaysia			Philippines			Thailand		
	Number	Percent	Rate	Number	Percent	Rate	Number	Percent	Rate
**Males and females**									
1995							0	0.0%	0.00
1996							3	0.1%	0.01
1997							1	0.0%	0.00
1998							2	0.0%	0.00
1999				3	0.2%	0.01	18	0.2%	0.04
2000	9	0.4%	0.06	3	0.1%	0.01	18	0.1%	0.04
2001	28	1.2%	0.16	10	0.2%	0.04			
2002	38	1.5%	0.24	7	0.1%	0.01	5	0.0%	0.01
2003	50	1.8%	0.30	20	0.4%	0.08	2	0.0%	0.00
2004	29	1.0%	0.18				3	0.0%	0.01
2005	49	1.7%	0.29				0	0.0%	0.00
2006	23	0.9%	0.15				1	0.0%	0.00
2007	46	1.8%	0.26						
2008	25	0.9%	0.13	7	0.1%	0.01			
**Males**									
1995							0	0.0%	0.00
1996							3	0.1%	0.02
1997							0	0.0%	0.00
1998							2	0.0%	0.01
1999				3	0.2%	0.01	13	0.1%	0.05
2000	7	0.4%	0.09	2	0.0%	0.01	12	0.1%	0.05
2001	25	1.3%	0.29	5	0.1%	0.03			
2002	29	1.4%	0.35	5	0.1%	0.02	2	0.0%	0.01
2003	43	2.0%	0.52	9	0.2%	0.10	2	0.0%	0.01
2004	27	1.2%	0.34				3	0.0%	0.01
2005	44	1.9%	0.52				0	0.0%	0.00
2006	19	0.9%	0.23				1	0.0%	0.00
2007	34	1.7%	0.37						
2008	18	0.8%	0.20	5	0.1%	0.02			
**Females**									
1995							0	0.0%	0.00
1996							0	0.0%	0.00
1997							1	0.1%	0.00
1998							0	0.0%	0.00
1999				0	0.0%	0.00	5	0.2%	0.02
2000	2	0.4%	0.02	1	0.1%	0.00	6	0.2%	0.03
2001	3	0.7%	0.03	5	0.4%	0.04			
2002	9	2.1%	0.11	2	0.2%	0.01	3	0.1%	0.01
2003	7	1.3%	0.07	11	0.8%	0.06	0	0.0%	0.00
2004	2	0.4%	0.03				0	0.0%	0.00
2005	5	0.9%	0.05				0	0.0%	0.00
2006	4	0.8%	0.07				0	0.0%	0.00
2007	12	2.3%	0.15						
2008	7	1.3%	0.07	2	0.1%	0.01			

### Joinpoint Regression Analysis

**Figure 3:**
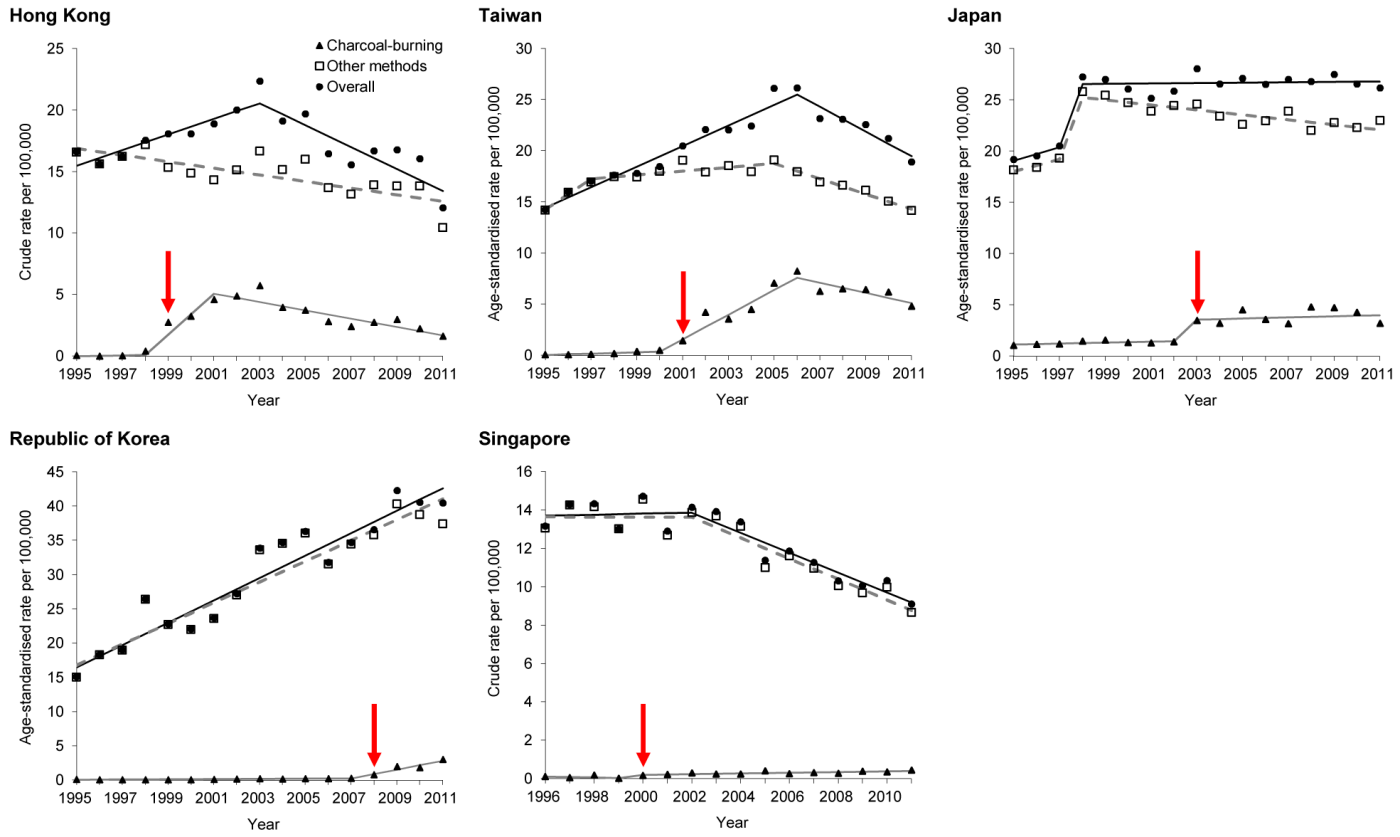
Time trends in suicide rates by method, with linear trends from joinpoint regression analysis. Red arrows indicate the years when charcoal-burning suicides started to increase.

Joinpoint regression analysis showed that the rise in charcoal-burning suicide began after 1998 (95% CI 1997–1999) in Hong Kong, 1999 (95% CI 1998–2001) in Singapore, 2000 (95% CI 1999–2001) in Taiwan, 2002 (95% CI 1999–2003) in Japan, and 2007 (95% CI 2006–2008) in the Republic of Korea (Table 3; Figure 3; see Figure S2 for method-specific graphs). Changes in time trends in Singapore were less certain, as they were mostly driven by the zero value in 1999; furthermore, a sensitivity analysis based only on certified suicides showed a continuously upward trend in the Singaporean charcoal-burning suicide rate over the entire study period, without any changes in time trends (Figure S3; Table S2). Analysis using Japanese data for 1995–2007 (excluding data for 2008–2010, when there was a rapid increase in suicide by hydrogen sulphide poisoning [31]) showed the same join points. Sensitivity analyses incorporating autocorrelation showed similar findings in all of the five countries (Table S3).

**Table 3:** Summary of the mean annual increases in suicide rate per 100,000 and join points for time trends in five East/Southeast Asian countries, 1995–2011.

Country	Suicide Method	Segment 1^a^		Join Point 1		Segment 2^a^		Join Point 2		Segment 3^a^	
		β	95% CI	Year	95% CI	β	95% CI	Year	95% CI	β	95% CI
**Hong Kong**	Charcoal-burning suicide	0.03	−0.12, 0.19	1998	1997, 1999	1.67	0.19, 3.14	2001	1999, 2004	−0.34	−0.45, −0.23
	Suicide by other methods	−0.27	−0.38, −0.16								
	Overall suicide	0.63	0.26, 1.01	2003	2001, 2005	−0.89	−1.23, −0.55				
**Taiwan**	Charcoal-burning suicide	0.07	−0.02, 0.15	2000	1999, 2001	1.21	0.83, 1.58	2006	2004, 2008	−0.50	−0.97, −0.02
	Suicide by other methods	1.51	0.26, 2.76	1997	1997, 2002	0.18	0.02, 0.35	2005	2003, 2007	−0.74	−0.93, −0.56
	Overall suicide	1.01	0.85, 1.16	2006	2005, 2007	−1.21	−1.70, −0.72				
**Japan**	Charcoal-burning suicide	0.05	−0.07, 0.16	2002	1999, 2003	2.10	—b	2003	2002, 2009	0.05	−0.12, 0.23
	Suicide by other methods	0.58	−0.89, 2.05	1997	1997, 1998	6.06	—b	1998	1998, 1999	−0.24	−0.33, −0.15
	Overall suicide	0.66	−1.08, 2.41	1997	1997, 1998	6.14	—b	1998	1998, 1999	0.02	−0.09, 0.13
**Republic of Korea**	Charcoal-burning suicide	0.02	0.01, 0.03	2007	2006, 2008	0.65	0.49, 0.82				
	Suicide by other methods	1.52	1.26, 1.77								
	Overall suicide	0.65	0.41, 0.90								
**Singapore**	Charcoal-burning suicide	−0.03	−0.10, 0.04	1999	1998, 2001	0.18	—b	2000	1999, 2006	0.02	0.01, 0.03
	Suicide by other methods	0.00	−0.35, 0.35	2002	1998, 2005	−0.54	−0.70, −0.38				
	Overall suicide	0.03	−0.32, 0.37	2002	1998, 2005	−0.52	−0.68, −0.36				

Rates per 100,000 are age-standardised rates for Taiwan, Japan, and the Republic of Korea, and crude rates for Hong Kong and Singapore. Data for Singapore are for 1996–2011.

a Segments were linear trends between join points (i.e., the years when the trends changed) identified using joinpoint regression, which characterises time trends as contiguous linear segments and join points.

b 95% CI could not be estimated by the joinpoint regression as the segment included only two data points.

β, mean annual increase in suicide rate per 100,000.

If the year following the join point when the time trend turned upward is defined as the year when the increase in charcoal-burning suicide began (indicated by arrows in Figure 3), Hong Kong first experienced a rise in charcoal-burning suicide in 1999, followed by Singapore in 2000, Taiwan in 2001, Japan in 2003, and the Republic of Korea in 2008. Joinpoint analysis showed that the upward trend in charcoal-burning suicide changed and turned downward around 2001 (95% CI 1999–2004) in Hong Kong and 2006 (95% CI 2004–2008) in Taiwan (Table 3; Figure 3), although inspection of time trends showed that Hong Kong's charcoal-burning suicide rate started to fall only after 2003 (Table 1; Figure 2). Similar declines in other methods of suicide in Hong Kong and Taiwan were also observed after 2003.

Combined numbers of charcoal-burning suicides for the five study countries reached a peak in 2009 (*n* = 6,759). When we excluded data for 2008–2010, because in Japan these may have included not only charcoal-burning suicides but also some suicides by hydrogen sulphide poisoning [31], the peak occurred in 2005 (*n* = 6,267). Compared to the baseline levels in the years before charcoal-burning suicide began to increase in individual countries (1998 for Hong Kong, 1999 for Singapore, 2000 for Taiwan, 2002 for Japan, and 2007 for the Republic of Korea), by 2011 there were 49,738 more charcoal-burning suicides in total across the region. The distribution of these cases was 32,636 in Japan (2003–2011), 11,306 in Taiwan (2001–2011), 2,506 in Hong Kong (1999–2011), 3,127 in the Republic of Korea (2008–2011), and 163 in Singapore (1999–2011), corresponding to an annual excess of 3,626, 1,028, 193, 782, and 14 suicides, respectively.

### Association with Overall Suicide Rates

Graphical examinations of time trends in suicide are presented in Figure 2. Table 3 and Figure 3 present joinpoint regression analyses. In Hong Kong, the rise in charcoal-burning suicide rate was associated with an increase in overall suicide rate over the period 1998–2003. Similarly, in Taiwan, the rise in charcoal-burning suicide in 2000–2006 was related to an increase in overall suicide rate over the same period. Some evidence of method substitution was found in Japanese males—the rise in charcoal-burning suicide was accompanied by a fall in suicide by other methods (Figure 2). In Japanese females, the increase in charcoal-burning suicide starting from 2003 was followed by an increase in the overall suicide rate, suggesting that the rise in charcoal-burning suicide may have contributed to the rise in the overall rate. In the Republic of Korea, the increase in charcoal-burning suicide was quite recent and was not associated with a rise in the overall suicide rate, as the magnitude of increase was relatively small. There was no evidence for an association of time trends in the rate of charcoal-burning suicide with changes in the overall suicide rate in Singapore.

### Sex- and Age-Specific Patterns

**Figure 4:**
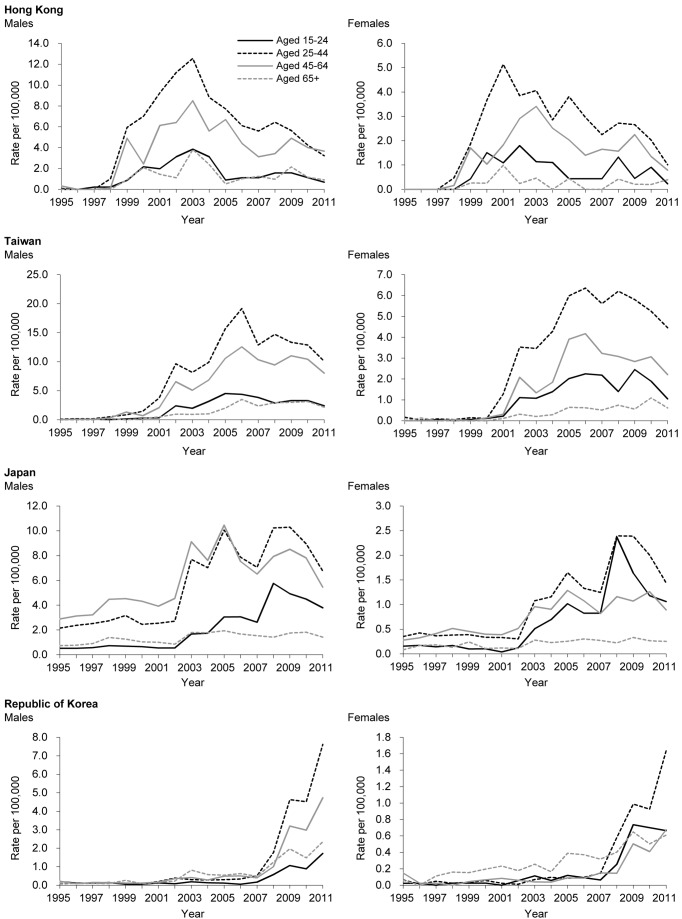
Sex- and age-specific time trends in charcoal-burning suicide in Hong Kong, Taiwan, Japan, and the Republic of Korea.

Figure 4 shows sex- and age-specific time trends in charcoal-burning suicide in Hong Kong, Taiwan, Japan, and the Republic of Korea. Males aged 25–44 y tended to show the highest rates compared to other sex/age groups, except that Japanese males aged 45–64 y had rates similar to those of males aged 25–44 y. In contrast, females aged 65+ y tended to have the lowest rates, except in the Republic of Korea, where females aged 45–64 y had the lowest rates. Negative binomial regression analysis showed that there was no statistically significant age or sex interaction in Hong Kong, Taiwan, Japan, or the Republic of Korea (*p* for interaction >0.05) (Table 4). Although the overall age interaction effects were not statistically significant, in Japan, males and females aged 15–24 y tended to have the greatest rates of increase (*p* for interaction = 0.064), whilst in the Republic of Korea, males and females aged 25–44 y and 45–64 y tended to have the greatest rates of increase (*p* for interaction = 0.096). When charcoal-burning suicide rates decreased in Hong Kong and Taiwan, there was no statistical evidence of sex or age differences in the annual rates of reduction (*p* for interaction = 0.53–0.86).

**Table 4:** Year-on-year incidence rate ratio for charcoal-burning suicide in four East/Southeast Asian countries.

Country	Age Group	Males and Females		Males		Females		*p* for Interaction	
		IRR	95% CI	IRR	95% CI	IRR	95% CI	Sex×Year	Age×Year
**Time periods when charcoal-burning suicide rates increased**									
Hong Kong (1998–2001)								0.75	0.87
	All ages	1.95	1.47, 2.58	1.85	1.38, 2.48	1.82	1.50, 2.20		
	Aged 15–24 y	1.98	1.18, 3.32	1.73	1.16, 2.59	1.97	0.96, 4.06		
	Aged 25–44 y	1.91	1.35, 2.70	1.81	1.22, 2.68	1.88	1.48, 2.40		
	Aged 45–64 y	1.84	0.82, 4.14	1.91	0.79, 4.59	1.49	0.96, 2.31		
	Aged 65+ y	2.54	2.54, 2.54	1.68	0.91, 3.12	2.68	0.96, 7.46		
Taiwan (2000–2006)								0.66	0.96
	All ages	1.49	1.37, 1.61	1.46	1.36, 1.57	1.53	1.37, 1.71		
	Aged 15–24 y	1.54	1.29, 1.85	1.53	1.27, 1.83	1.45	1.26, 1.69		
	Aged 25–44 y	1.45	1.25, 1.68	1.42	1.25, 1.61	1.53	1.18, 1.99		
	Aged 45–64 y	1.50	1.27, 1.77	1.46	1.26, 1.71	1.57	1.29, 1.93		
	Aged 65+ y	1.47	1.32, 1.64	1.49	1.33, 1.68	1.44	1.13, 1.84		
Japan (2002–2005)^a^								0.37	0.064
	All ages	1.40	1.25, 1.58	1.38	1.24, 1.55	1.46	1.28, 1.66		
	Aged 15–24 y	1.68	1.36, 2.08	1.63	1.33, 2.00	1.74	1.38, 2.19		
	Aged 25–44 y	1.46	1.15, 1.86	1.44	1.13, 1.83	1.62	1.24, 2.12		
	Aged 45–64 y	1.26	1.07, 1.48	1.25	1.06, 1.48	1.29	1.15, 1.46		
	Aged 65+ y	1.26	1.04, 1.52	1.26	1.06, 1.50	1.21	0.97, 1.51		
Republic of Korea (2007–2010)								0.22	0.096
	All ages	1.60	1.42, 1.81	1.64	1.45, 1.85	1.44	1.29, 1.62		
	Aged 15–24 y	1.59	1.27, 1.99	1.54	1.29, 1.84	1.57	1.13, 2.18		
	Aged 25–44 y	1.80	1.43, 2.28	1.84	1.45, 2.33	1.61	1.31, 1.97		
	Aged 45–64 y	1.74	1.41, 2.15	1.79	1.42, 2.25	1.46	1.22, 1.74		
	Aged 65+ y	1.27	1.10, 1.46	1.33	1.12, 1.57	1.15	0.97, 1.35		
**Time periods when charcoal-burning suicide rates decreased**									
Hong Kong (2003–2011)								0.53	0.67
	All ages	0.89	0.86, 0.91	0.89	0.86, 0.91	0.90	0.87, 0.93		
	Aged 15–24 y	0.87	0.79, 0.96	0.84	0.76, 0.94	0.93	0.80, 1.07		
	Aged 25–44 y	0.88	0.85, 0.90	0.87	0.84, 0.90	0.90	0.86, 0.94		
	Aged 45–64 y	0.90	0.86, 0.95	0.91	0.87, 0.96	0.89	0.84, 0.94		
	Aged 65+ y	0.91	0.82, 1.01	0.89	0.79, 1.00	1.03	0.81, 1.31		
Taiwan (2006–2011)								0.86	0.55
	All ages	0.93	0.90, 0.95	0.93	0.90, 0.95	0.94	0.91, 0.96		
	Aged 15–24 y	0.91	0.86, 0.97	0.91	0.85, 0.97	0.91	0.82, 1.02		
	Aged 25–44 y	0.92	0.88, 0.96	0.91	0.87, 0.95	0.95	0.92, 0.98		
	Aged 45–64 y	0.93	0.90, 0.97	0.94	0.90, 0.99	0.91	0.86, 0.95		
	Aged 65+ y	0.97	0.91, 1.05	0.96	0.88, 1.04	1.06	0.90, 1.25		

a The peak year (2005) for Japan was determined by inspecting the time trends to identify the year when charcoal-burning suicide rate was the highest before 2008, as from 2008 onwards suicides classified as other gas poisoning included not only charcoal-burning suicides but also some deaths from hydrogen sulphide poisoning [31].

IRR, incidence rate ratio.

## Discussion

### Main Findings

Charcoal-burning suicides increased markedly in some East/Southeast Asian countries (Hong Kong, Taiwan, Japan, the Republic of Korea, and Singapore) in the first decade of the 21st century, but such rises were not experienced by all countries in the region. In countries with a rise in the charcoal-burning suicide rate, the timing, scale, and sex/age pattern of the increase varied by country. A rise in charcoal-burning suicides was first seen in Hong Kong (1999), followed by Singapore (2000), Taiwan (2001), Japan (2003), and the Republic of Korea (2008), although the evidence for a definite starting year for Singapore was limited because of relatively small suicide numbers. Evidence for an association between the charcoal-burning suicide rate and the overall suicide rate varied by country—an association was found in Hong Kong, Taiwan, and Japan (for females), but not in Japan (for males), the Republic of Korea, and Singapore. Combined numbers of charcoal-burning suicides for the five study countries reached a peak in 2009, resulting in around 6,800 deaths. Compared to the baseline levels prior to the increase in charcoal-burning suicide in individual countries, by 2011 there were nearly 50,000 excess suicides by charcoal burning in total. Annual rates of changes in charcoal-burning suicide rates did not differ by sex/age group in Taiwan and Hong Kong, whilst people aged 15–24 y in Japan and people aged 25–64 y in the Republic of Korea tended to have the greatest rates of increase.

Our data showed that the increases in charcoal-burning suicide were associated with various levels of changes in overall suicide rates across the East/Southeast Asian countries studied. The increase in charcoal-burning suicide first started in Hong Kong and Taiwan, where it was associated with a rise in overall suicide rates, followed by an increase in Japan, where the rise in overall suicide was less obvious, and then in the Republic of Korea, where the increase in charcoal-burning suicide was very recent, and further investigation of its association with any changes in overall suicide rate is needed. In contrast, Singapore had a much smaller rise in charcoal-burning suicide than other countries did.

### Strengths and Limitations

To the best of our knowledge, this is the first systematic investigation of the rise in the popularity of charcoal burning as a method of suicide across East/Southeast Asian countries. There are several limitations to this study. First, we were not able to differentiate charcoal-burning suicides from deaths using other sources of non-domestic gas, such as car exhaust fumes or hydrogen sulphide. However, previous studies found that charcoal-burning suicide accounted for over 90% of all suicides from non-domestic gas poisoning in Hong Kong [10] and Taiwan [30], and there has been no report of a prominent rise in gassing suicide using car exhaust fumes in the East/Southeast Asian countries studied. One recent investigation of Japanese suicide data collected by the police showed that there were 2,726 charcoal-burning suicides in 2007, accounting for 8.2% of all suicides [29]; this finding is similar to our results based on certified suicides coded as poisoning using all non-domestic gases (3,044 charcoal-burning suicides, accounting for 9.9% of all suicides). A marked rise in suicide by hydrogen sulphide poisoning may have accounted for part of the increase in suicide by non-domestic gas poisoning in Japan during the time period 2008–2010 [31],[48]; however, the rise in charcoal-burning suicide in 2003 was 5 y before the increase in hydrogen sulphide suicide and was consistent with previous reports of the emergence of charcoal-burning suicide in Japan [14].

Second, the quality of suicide data may vary by country and change over time. One important factor of under-reporting is the misclassification of suicides as deaths of other causes, such as death of undetermined intent [30],[32]–[35]. Our main analyses included both suicides and deaths coded as undetermined intent, and findings were similar when data only for certified suicides were used. In addition, death investigation systems, procedures, and practices differ across countries; this may have influenced the reliability of suicide statistics. In Hong Kong and Singapore, which both follow the British common law system, the cause of death for all unnatural deaths is determined by the coroner's court based on evidence collected by the police [49],[50]. However, the use of codes of undetermined intent appeared to be more common in Singapore than in Hong Kong (the undetermined death/suicide ratio was 0.02 in Hong Kong and 0.31 in Singapore in 2011), suggesting greater under-reporting of suicide in Singapore. In Taiwan, Japan, and the Republic of Korea, which all use the continental law system, there is no coroner's inquest for suicide, but all unnatural deaths have to undergo a medico-legal investigation to rule out the possibility of homicide [24],[32]. Suicide estimates in these five countries are considered to be reliable according to the rating scheme of the WHO [24]. Suicide statistics are subject to under-reporting and misclassification in Malaysia, the Philippines, and Thailand, where the quality of suicide registration is not satisfactory [24]–[27].

Third, we did not include data for some East/Southeast Asian countries where cases of charcoal-burning suicide were also reported recently, such as China [13] and Macao [15]; detailed method-specific data for suicide were unavailable for these countries. Last, we did not include any covariates in our models to investigate other factors that may have influenced suicide rates. However, the main aim of this study was to identify changes in charcoal-burning suicide rates and their associations with overall suicide rates; the simple linear trend models used in this study, i.e., joinpoint regression and negative binomial models, are appropriate for this purpose.

### Possible Explanations of International Variations

Several factors may have contributed to differences in the timing, scale, and sex/age pattern of rises in charcoal-burning suicide amongst East/Southeast Asian countries. How and the extent to which the media reported the first or first few cases of charcoal-burning suicide may have played an important role in the adoption pattern of the method. It has been reported that extensive media reporting of cases was followed by an increase in charcoal-burning suicide in Hong Kong [21],[22], Taiwan [51], Japan [52],[53], and the Republic of Korea [54]. Hong Kong and Taiwan share the same written language (traditional Chinese characters), and media reports of charcoal-burning suicide in one country could be easily understood by people living in the other country. In Macao and Singapore, where traditional Chinese characters are also commonly used, emerging cases of charcoal-burning suicide were reported as early as 2000–2001 [15],[17]. In contrast, Japan and the Republic of Korea use very different languages, and charcoal-burning suicides emerged some years later than those in the aforementioned Chinese societies. Malaysia, the Philippines, and Thailand also use different languages. In Chinese populations, it is commonly believed that preserving the appearance of the body is important for a better next life [21], and in Japan, death with a “peaceful face” is considered a good death [55]; these considerations may have made charcoal burning an appealing suicide method in these populations.

Variations in familiarity with coal or charcoal and their accessibility are also possible contributors to the difference between countries in the uptake of this suicide method. In Hong Kong and Taiwan, barbecue charcoal is readily accessible at local shops; such easy availability and familiarity with barbecue charcoal may have contributed to the rapid increases in charcoal-burning suicide in these countries. In contrast, charcoal-burning suicides were usually referred to in Japan as suicides using “rentan” [56], which was a general term for different kinds of fuels made of coal or charcoal, including the old-fashioned coal briquette, which is not readily available nowadays in Japan [57]. Similarly in the Republic of Korea, the highest profile reported case of charcoal burning used an equivalent rentan-type coal briquette (“yeontan”). The availability of these briquettes has declined in both Japan and the Republic of Korea over the last two decades [58],[59]. Overall, there seems to be no evidence of a rapid increase in the availability of barbecue charcoal in the countries we studied over the past decade.

In Hong Kong, charcoal-burning suicides emerged in 1998–1999, following the Asian economic crisis in 1997–1998, which was shown to have a strong impact on Hong Kong's economy and suicide patterns [39]. The economic recession, which led to an increased number of people troubled by debt problems, may have had some role in the increase in charcoal-burning suicide [22]. However, although the Asian economic crisis was also associated with a rise in overall suicide rates in Japan and the Republic of Korea [39], rises in charcoal-burning suicide did not occur in these two countries until several years later. In Taiwan, the rise and fall of charcoal-burning suicide did not seem to be associated with economic conditions [60],[61]. Furthermore, although economic slowdowns may be accompanied by rises in suicide [39], the impact is not likely to be method-specific (i.e., affecting only trends in charcoal-burning suicide but not suicide using other methods).

Our data showed that—for both the time periods when charcoal-burning suicide rates rose and fell—the rate of change did not differ by sex or age group in Taiwan or Hong Kong. In contrast, young males and females in Japan as well as middle-aged males and young females in the Republic of Korea appeared to have the most rapid increase in charcoal-burning suicide compared to other age groups, although the statistical evidence for age interaction was not significant (*p* for interaction = 0.064–0.096). This may be related to the characteristics of the initial cases. For example in Japan, young people and teenagers were involved in several widely reported suicide pacts of charcoal-burning suicide [53],[62],[63].

### Implications

Our results have several implications for international and regional suicide prevention strategies. First, our findings indicate that it is important to undertake surveillance to identify the emergence of new suicide methods. Such surveillance may include investigating the characteristics of suicide attempts in which such methods are first adopted, and the channels through which information about the new method spreads. Such information will help identify potential measures that may prevent or stop the use of new dangerous methods at an early stage. Second, it is crucial to work with the media and policy makers to restrict graphic descriptions of novel suicide methods and technical information about how to use them. Similarly, it is also important to work with internet service providers to regulate online content containing the details of dangerous methods, which may contribute to the increasing use of new suicide methods not only within but also across country boundaries. Both such measures will limit the cognitive availability of new, high-lethality methods [64]. A recent study of charcoal-burning suicides in England showed that over one-third of the individuals obtained information about the method from the internet [65]. Third, because of cultural similarity and geographical closeness, charcoal-burning suicide may become popular in other East/Southeast Asian countries where charcoal-burning suicide is currently rare. Related agencies and researchers in this area should be mindful of this possibility; they should share and exchange information about surveillance and appropriate policy responses to newly emerging suicide methods. Finally, in areas where the new methods are already widely used, we should consider a range of intervention strategies such as means restriction [66], gate keeper training [67], and improving mental health and help seeking in high-risk groups [68].

## Supporting Information

Alternative Language Abstract S1 **Simplified Chinese translation of the abstract by SSC.**
(DOCX)

Alternative Language Abstract S2 **Traditional Chinese translation of the abstract by SSC.**
(DOCX)

Alternative Language Abstract S3 **Japanese translation of the abstract by AH.**
(RTF)

Alternative Language Abstract S4 **Korean translation of the abstract by WJL.**
(DOCX)

Figure S1 **Time trends in suicide rates: overall suicide, charcoal-burning suicide, and suicide by other methods (using certified suicide cases).**
(TIF)

Figure S2 **Time trends in suicide rates: charcoalburning suicide, suicide by other methods, and overall suicide, with linear trends from joinpoint regression analysis.** Arrows indicate the years when charcoal-burning suicides started to increase.
(TIF)

Figure S3 **Time trends in suicide rates by method, with linear trends from joinpoint regression analysis (using certified suicide cases).** Arrows indicate the years when charcoal-burning suicides started to increase.
(TIF)

Table S1 **Number, percent, and rate per 100,000 of charcoal-burning suicide (based on certified suicide cases only).**
(DOC)

Table S2 **Summary of the mean annual increases in suicide rate (based on certified suicide cases only) per 100,000 and join points for time trends in five East/ Southeast Asian countries, 1995–2011.**
(DOC)

Table S3 **Summary of the mean annual increases in suicide rate per 100,000 and join points for time trends, accounting for autocorrelation, in five East/Southeast Asian countries, 1995–2011.**
(DOC)

## Abbreviations

ICD: International Classification of Diseases
ICD-9: ninth revision of International Classification of Diseases
ICD-10: tenth revision of International Classification of Diseases
WHO: World Health Organization
