# Supplementary material for: Regional Changes in Charcoal-Burning Suicide Rates in East/Southeast Asia from 1995 to 2011: A Time Trend Analysis
Source: PLoS Med. 2014 Apr 1;11(4):e1001622. doi: 10.1371/journal.pmed.1001622 (PMC3972087; doi:10.1371/journal.pmed.1001622)
Supplement: Alternative Language Abstract S1 — Simplified Chinese translation of the abstract by SSC. (DOCX) [file pmed.1001622.s001.docx]

# Translation of the Abstract into Simplified Chinese by Shu-Sen Chang

# 标题：东亚与东南亚国家1995-2011年间的烧炭自杀率变化：时间趋势分析

摘要（Abstract）

背景（Background）

在二十一世纪初期，烧木炭导致一氧化碳中毒而自杀身亡的首例个案发生后的短短五年间，以同样方法自杀身亡的个案在香港与台湾快速增加。本研究分析：（1）1995-2011年间东亚与东南亚国家烧炭自杀率的趋势变化；（2）是否烧炭自杀率的增加伴随着总体自杀率的增加。我们同时也分析不同性别/年龄的趋势变化，找出在不同国家烧炭自杀率增加最显著的族群。

研究方法与发现（Methods and Findings）

我们分析香港、日本、南韩、台湾和新加坡（主要研究国家）在1995/1996到2011年间，使用非家用瓦斯而自杀身亡个案的资料，相较下，马来西亚、菲律宾和泰国的数据年份较不完整。我们使用趋势图和转折点回归分析，分析自杀率的时间趋势，并用负二项回归模型研究不同性别/年龄的差异。在1995/1996年，在主要研究国家烧炭自杀仅占所有自杀少于百分之一，除了日本之外（百分之五），然而，在2011年在香港、台湾、日本、南韩和新加坡分别增加到百分之十三、二十四、十、七和五。烧炭自杀之增加，首见于香港（1998年后，百分之九十五信赖区间为1997-1999年），接续为新加坡（1999年后，1998-2001年）、台湾（2000年后，1999-2001年）、日本（2002年后，1999-2003年）、与南韩（2007年后，2006-2008年）。在马来西亚、菲律宾和泰国则未见到烧炭自杀显著增加。有证据显示在香港、台湾与日本（女性），烧炭自杀的增加伴随着总体自杀率的增加，但在日本（男性）、南韩和新加坡则未见此现象。烧炭自杀的增加率，在台湾和香港未有性别/年龄间的差异，但是日本以十五岁到二十四岁族群最为显著，在南韩则是二十五岁到六十四岁族群最为显著。本研究的限制之一，是各国所使用的国际疾病分类手册中并无烧炭自杀的专用分类码，同时各国的死因编码方式可能有差异。

结论（Conclusions）

在二十一世纪的最初十年间，在某些东亚与东南亚国家，烧炭自杀率显著增加，但并非所有此地区的国家都发生相同现象。在烧炭自杀率增加的国家，其增加的起始年份、规模和性别年龄差异有所不同，需要进一步研究了解造成这些变异的原因，例如文化和媒体报导方式的差异。
