# Supplementary material for: Regional Changes in Charcoal-Burning Suicide Rates in East/Southeast Asia from 1995 to 2011: A Time Trend Analysis
Source: PLoS Med. 2014 Apr 1;11(4):e1001622. doi: 10.1371/journal.pmed.1001622 (PMC3972087; doi:10.1371/journal.pmed.1001622)
Supplement: Alternative Language Abstract S2 — Traditional Chinese translation of the abstract by SSC. (DOCX) [file pmed.1001622.s002.docx]

# Translation of the Abstract into Complex Chinese by Shu-Sen Chang

# 標題：東亞與東南亞國家1995-2011年間的燒炭自殺率變化：時間趨勢分析

摘要（Abstract）

背景（Background）

在二十一世紀初期，燒木炭導致一氧化碳中毒而自殺身亡的首例個案發生後的短短五年間，以同樣方法自殺身亡的個案在香港與台灣快速增加。本研究分析：（1）1995-2011年間東亞與東南亞國家燒炭自殺率的趨勢變化；（2）是否燒炭自殺率的增加伴隨著總體自殺率的增加。我們同時也分析不同性別/年齡的趨勢變化，找出在不同國家燒炭自殺率增加最顯著的族群。

研究方法與發現（Methods and Findings）

我們分析香港、日本、南韓、台灣和新加坡（主要研究國家）在1995/1996到2011年間，使用非家用瓦斯而自殺身亡個案的資料，相較下，馬來西亞、菲律賓和泰國的資料年份較不完整。我們使用趨勢圖和轉折點迴歸分析，分析自殺率的時間趨勢，並用負二項迴歸模型研究不同性別/年齡的差異。在1995/1996年，在主要研究國家燒炭自殺僅占所有自殺少於百分之一，除了日本之外（百分之五），然而，在2011年在香港、台灣、日本、南韓和新加坡分別增加到百分之十三、二十四、十、七和五。燒炭自殺之增加，首見於香港（1998年後，百分之九十五信賴區間為1997-1999年），接續為新加坡（1999年後，1998-2001年）、台灣（2000年後，1999-2001年）、日本（2002年後，1999-2003年）、與南韓（2007年後，2006-2008年）。在馬來西亞、菲律賓和泰國則未見到燒炭自殺顯著增加。有證據顯示在香港、台灣與日本（女性），燒炭自殺的增加伴隨著總體自殺率的增加，但在日本（男性）、南韓和新加坡則未見此現象。燒炭自殺的增加率，在台灣和香港未有性別/年齡間的差異，但是日本以十五歲到二十四歲族群最為顯著，在南韓則是二十五歲到六十四歲族群最為顯著。本研究的限制之一，是各國所使用的國際疾病分類手冊中並無燒炭自殺的專用分類碼，同時各國的死因編碼方式可能有差異。

結論（Conclusions）

在二十一世紀的最初十年間，在某些東亞與東南亞國家，燒炭自殺率顯著增加，但並非所有此地區的國家都發生相同現象。在燒炭自殺率增加的國家，其增加的起始年份、規模和性別年齡差異有所不同，需要進一步研究了解造成這些變異的原因，例如文化和媒體報導方式的差異。
