# Supplementary material for: Regional Changes in Charcoal-Burning Suicide Rates in East/Southeast Asia from 1995 to 2011: A Time Trend Analysis
Source: PLoS Med. 2014 Apr 1;11(4):e1001622. doi: 10.1371/journal.pmed.1001622 (PMC3972087; doi:10.1371/journal.pmed.1001622)
Supplement: Alternative Language Abstract S3 — Japanese translation of the abstract by AH. (RTF) [file pmed.1001622.s003.rtf]

表\題è:1995年Nか©らç2011年NまÜでÅのÌ東Œ・E東Œ南ìアAジWアAにÉお¨け¯るé練û炭Y自©殺E率¦のÌ地n域æ変Ï化»：F時ž系n列ñ解ð析Í

著˜者Ò: Shu-Sen Chang a,b,c,d, Ying-Yeh Chen e,f,*, Paul S.F. Yip a,b, Won Jin Lee g, Akihito Hagihara h, David Gunnell d

所Š属®: 
a HKJC Centre for Suicide Research and Prevention, The University of Hong Kong, 5 Sassoon Road, Pokfulam, Hong Kong SAR, China; 
b Department of Social Work and Social Administration, The University of Hong Kong, Pokfulam Road, Pokfulam, Hong Kong SAR, China;
c Ju Shan Hospital, No 910, Daguan Road, Dayuan Township, Taoyuan 337, Taiwan; 
d School of Social and Community Medicine, University of Bristol, Canynge Hall, 39 Whatley Road, Bristol BS8 2PS, United Kingdom; 
e Taipei City Psychiatric Center, Taipei City Hospital, Taipei, 309 Songde Road, XinYi District, Taipei City 110, Taiwan; 
f Institute of Public Health and Department of Public Health, National Yang-Ming University, No 155, Sec 2, Linong Street, Beitou District, Taipei City 112, Taiwan; 
g Department of Preventive Medicine, College of Medicine, Korea University, 
Seoul 136-705, Republic of Korea;
h Department of Health Services Management and Policy, Kyushu University Graduate School of Medicine, 3-1-1 Higashiku, Fukuoka 812-8582, Japan

*連A絡�先æ：FYing-Yeh Chen, 309 Songde Road, XinYi District, Taipei City 110, Taiwan (email: ychen@tpech.gov.tw; fax: +886 227272150; tel: +886 227263141 ext. 1347)


要v旨|
背w景i: 
2000年N代ãのÌ初‰頭ªにÉバoー[ベxキLュ…ー[用pのÌ炭YでÅ一ê酸_化»炭Y素fをð発­生¶さ³せ¹自©殺E（i練û炭Y自©殺E）jしµた½最Å初‰のÌ事–例áがª報ñ告�さ³れêてÄか©らç、A5年Nもà経oた½なÈい¢内àにÉ練û炭Y自©殺Eがª香�港`とÆ台ä湾pでÅ大å流¬行sしµた½。B本{研¤究†のÌ目Ú的IはÍ（i１P）j1995年Nか©らç2011年NまÜでÅのÌ東Œ・E東Œ南ìアAジWアAにÉお¨け¯るé練û炭Y自©殺E率¦のÌ時ž系n列ñ変Ï化»お¨よæびÑ地n域æ特Á性«のÌ検Ÿ討¢、A（i２Q）j練û炭Y自©殺E率¦とÆ全S体Ì的IなÈ自©殺E率¦のÌ増�加ÁとÆのÌ関Ö係WのÌ有L無³のÌ検Ÿ討¢、AでÅあ るé。B対Î象Û地n域æでÅ練û炭Y自©殺E率¦のÌ大å幅�なÈ上ã昇¸をð示¦しµた½グOル‹ー[プvをð特Á定èす·るéた½めß、A練û炭Y自©殺E率¦のÌ性«別Ê、A年N齢î別ÊのÌ動®向üもà併¹せ¹てÄ検Ÿ討¢しµた½。B 

方û法@とÆ知m見©:
香�港`、A日ú本{、A韓Ø国‘、A台ä湾p、AシVン“ガKポ|ー[ル‹のÌ1995なÈい¢しµ1996年Nか©らç2011年NのÌガKスXにÉよæるé自©殺EにÉ関Öす·るéデfー[タ^をð使g用pしµた½。Bマ}レŒー[シVアA、AフtィBリŠピsン“、Aタ^イCにÉつÂい¢てÄもà同¯様lのÌデfー[タ^をð収û集Wしµた½がª、A不s完®全SでÅあ っÁた½。BGraphical and joinpoint regression 分ª析Íをð用pい¢てÄ自©殺EのÌ時ž系n列ñ変Ï化»をð解ð析Íしµ、ANegative binomial regression 解ð析Íをð用pい¢てÄ性«別Ê、A年N齢î別ÊのÌパpタ^ー[ン“をð検Ÿ討¢しµた½。B1995年NまÜた½はÍ1996年NにÉ練û炭Y自©殺EはÍ日ú本{（i5%）j以È外OのÌ全SてÄのÌ国‘でÅ1% 以È下ºでÅあ っÁた½。Bしµか©しµ、A2011年NにÉはÍ、A香�港`、A台ä湾p、A日ú本{、A韓Ø国‘、AシVン“ガKポ|ー[ル‹でÅ、Aそ»れêぞ¼れê、A13%, 24%, 10%, 7%, お¨よæびÑ 5%にÉ増�加Áしµた½。B最Å初‰のÌ上ã昇¸はÍ1998年NにÉ香�港`でÅ見©らçれê (95% CI：F1997-1999)、A 続±い¢てÄ1999年NのÌシVン“ガKポ|ー[ル‹ (95% CI：F 1998-2001)、A2000年NのÌ台ä湾p (95% CI：F1999-2001)、A2002年NのÌ日ú本{ (95% CI: 1999-2003)、Aそ»しµてÄ、A2007年NのÌ韓Ø国‘ (95% CI 2006-2008)のÌ順‡でÅ見©らçれêた½。Bマ}レŒー[シVアA、AフtィBリŠピsン“、Aタ^イCでÅはÍ顕°著˜なÈ上ã昇¸はÍ見©らçれêなÈか©っÁた½。B香�港`、A台ä湾p、A日ú本{（i女—性«のÌみÝ）jでÅはÍ、A練û炭Y自©殺E率¦とÆ全S体Ì的IなÈ自©殺E率¦とÆのÌ間ÔにÉ関Ö連A性«がª見©らçれêた½がª、A日ú本{（i男j性«）j、A韓Ø国‘、AシVン“ガKポ|ー[ル‹でÅはÍ見©らçれêなÈか©っÁた½。B台ä湾p、A香�港`でÅはÍ性«別Ê、A年N齢î別ÊでÅ変Ï化»率¦にÉ差·はÍ見©らçれêなÈか©っÁた½がª、A日ú本{でÅはÍ15-24歳Î、A韓Ø国‘でÅはÍ25-64歳ÎでÅ変Ï化»率¦がª最Å大åにÉなÈっÁてÄい¢た½。B国‘際Û疾¾病a分ª類Þ(ICD)でÅはÍ練û炭Y自©殺EのÌ分ª類Þ項€目ÚがªなÈく­、A対Î象Û国‘でÅ分ª類Þ基î準€がª異ÙなÈっÁてÄい¢るéこ±とÆがª本{研¤究†のÌ限À界EでÅあ っÁた½。B 
結‹論_:
21世¢紀I最Å初‰のÌ10年N間ÔでÅ、A東Œ・E東Œ南ìアAジWアA地n域æ（i香�港`、A台ä湾p、A日ú本{、A韓Ø国‘、AシVン“ガKポ|ー[ル‹）jでÅ練û炭Y自©殺EのÌ顕°著˜なÈ増�加Áがª見©らçれêた½。Bしµか©しµ、Aこ±のÌよæう¤なÈ増�加ÁはÍ東Œ・E東Œ南ìアAジWアAのÌ全S域æでÅはÍ見©らçれêなÈか©っÁた½。B練û炭Y自©殺EのÌ増�加Áがª見©らçれêた½地n域æでÅはÍ、A増�加ÁのÌ時ž期ú、A規K模Í、A年N齢î別Ê、A性«別ÊのÌパpタ^ー[ン“がª異ÙなÈっÁてÄい¢た½。Bこ±れêらçバoラ‰ツcキLのÌ要v因öにÉ関ÖしµてÄはÍ更XなÈるé検Ÿ討¢がª必K要vでÅあ るéがª、A文¶化»お¨よæびÑ自©殺E方û法@にÉ関Öす·るéメ�デfイCアA報ñ道¹のÌ違áい¢がª関Ö連AしµてÄい¢るé可Â能\性«がªあ るé。B
キLイCワ�ー[ドh:自©殺E;　@時ž系n列ñ; 練û炭Y燃R焼Ä; 一ê酸_化»炭Y素f中†毒Å, 死€亡S, 疫u学w;　@香�港`; 台ä湾p; 日ú本{; 韓Ø国‘; シVン“ガKポ|ー[ル‹
