# Supplementary material for: Regional Changes in Charcoal-Burning Suicide Rates in East/Southeast Asia from 1995 to 2011: A Time Trend Analysis
Source: PLoS Med. 2014 Apr 1;11(4):e1001622. doi: 10.1371/journal.pmed.1001622 (PMC3972087; doi:10.1371/journal.pmed.1001622)
Supplement: Alternative Language Abstract S4 — Korean translation of the abstract by WJL. (DOCX) [file pmed.1001622.s004.docx]

동/동남아시아 지역에서 1995-2011년간 숯으로 인한 자살률의 지역별 변화: 시계열 분석

**배경:**

홍콩과 대만에서 숯으로 인한 일산화탄소 자살이 2000년대 초 처음 보고된 이후 5년 내에 유행수준에 도달하였다. 이 연구의 목적은 i) 동/동남아시아 지역에서 1995-2011년간 숯(charcoal-burning) 자살의 시간적 경향성과 지역적 양상을 파악하고 ii) 숯을 사용한 자살 방법의 증가가 전체 자살률 증가와 관련되었는지 평가하는 것이다. 또한 각 국가들에서 숯 자살이 가장 크게 증가한 인구학적 집단을 파악하기 위해서 시간에 따른 성 및 연령별 경향성을 조사하였다.

**방법과 결과:**

이 연구에서 사용된 자료는 홍콩, 일본, 한국, 대만, 싱가포르에서는 비가정내 가스로 인한 자살 자료를, 말레이시아, 필리핀, 태국에서는 이와 비슷하지만 불완전한 자살 자료를 사용하였다. 자살의 시간적 경향성을 조사하기 위해서는 그래프 및 Joinpoint 회귀분석이, 성과 연령별 양상을 조사하기 위해서는 음이항회귀분석이 사용되었다. 숯 자살은 1995-1996년에 전체 자살에서 차지하는 분율이 일본을 (5%) 제외하고 모든 나라에서 1% 미만이었으나 2011년에는 홍콩, 대만, 일본, 한국, 싱가포르에서 각각 13%, 24%, 10%, 7%, 5%를 차지하였다. 이러한 증가는 1998년 홍콩에서 처음 관찰되었고 (95% 신뢰구간 1997-1999), 뒤를 이어 1999년에 싱가포르 (95% 신뢰구간 1998-2001), 2000년 대만 (95% 신뢰구간 1999-2001), 2002년 일본 (95% 신뢰구간 1999-2003), 2007년에 한국 (95% 신뢰구간 2006-2008)에서 관찰되었다. 그러나 이러한 증가가 말레이시아, 필리핀, 태국에서는 관찰되지 않았다. 숯 자살은 홍콩, 대만, 일본(여성), 한국, 싱가포르에서 전체 자살률을 증가시켰다. 숯 자살률의 변화는 대만과 홍콩에서 성 및 연령별로 다르지 않았으나 일본에서는 15-24세, 한국에서는 25-64세 집단에서 가장 높게 나타났다. 숯 자살에 대한 국제표준분류의 부재와 각 나라별 적용의 차이는 연구의 제한점이다.

**결론:**

숯 자살은 일부 동/동남아시아 국가들 (홍콩, 대만, 일본, 한국, 싱가포르)에서 21세기 초에 크게 증가하였으나 지역 내 전체 국가들에서 관찰되지는 않았다. 숯 자살이 증가된 각 국가들 내에서도 증가의 시기, 규모, 성 및 연령별 양상에 차이를 보였다. 따라서 이러한 차이와 관련된 요인들, 특히 자살 방법에 대한 문화적 차이 혹은 대중 매체의 보도 등과 관련된 추후 연구가 필요하다.
