# Supplementary figures and images for: Regional Changes in Charcoal-Burning Suicide Rates in East/Southeast Asia from 1995 to 2011: A Time Trend Analysis
Source: PLoS Med. 2014 Apr 1;11(4):e1001622. doi: 10.1371/journal.pmed.1001622 (PMC3972087; doi:10.1371/journal.pmed.1001622)

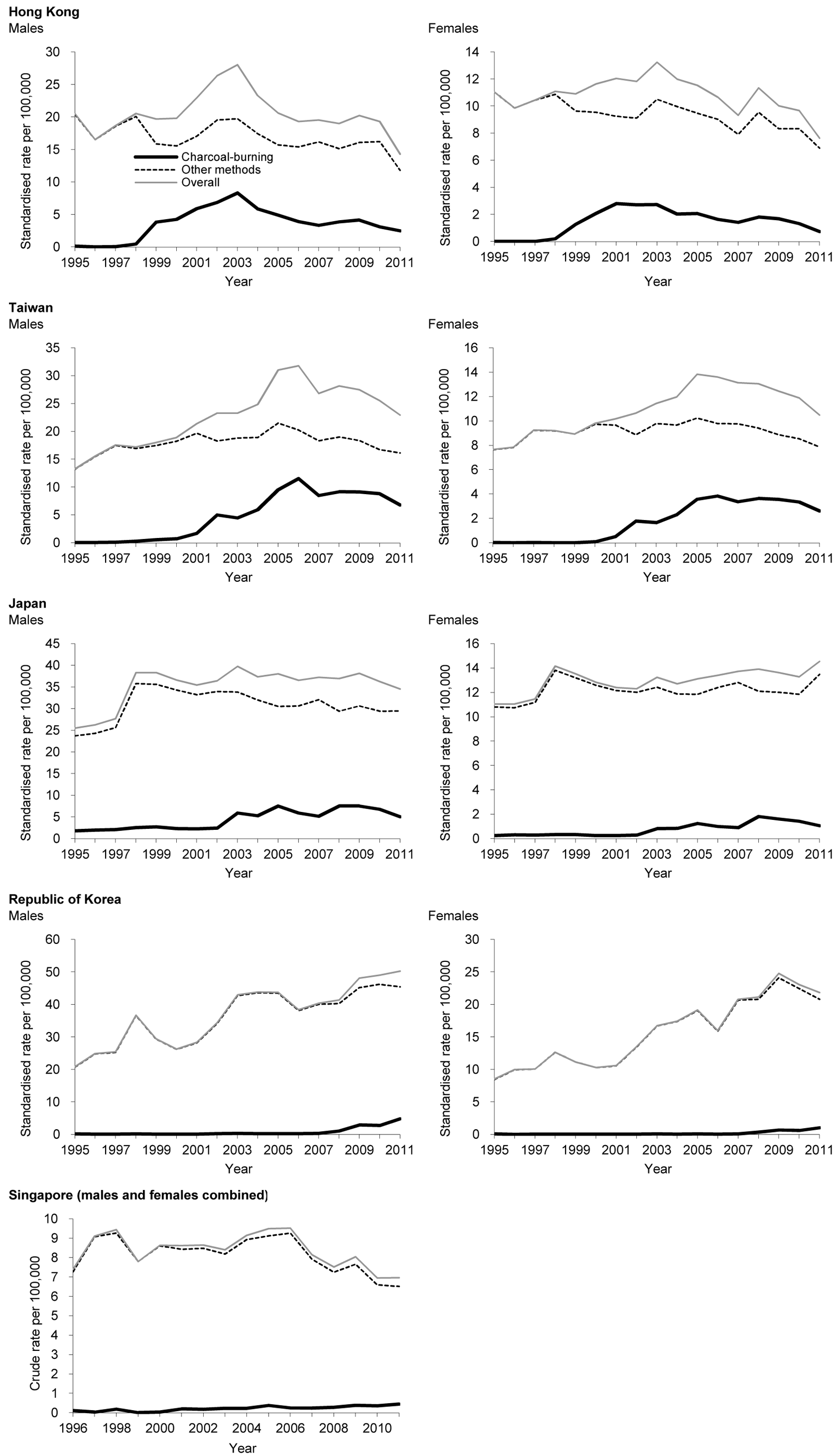

Supplement: Figure S1 — Time trends in suicide rates: overall suicide, charcoal-burning suicide, and suicide by other methods (using certified suicide cases). (TIF) [file pmed.1001622.s005.tif]

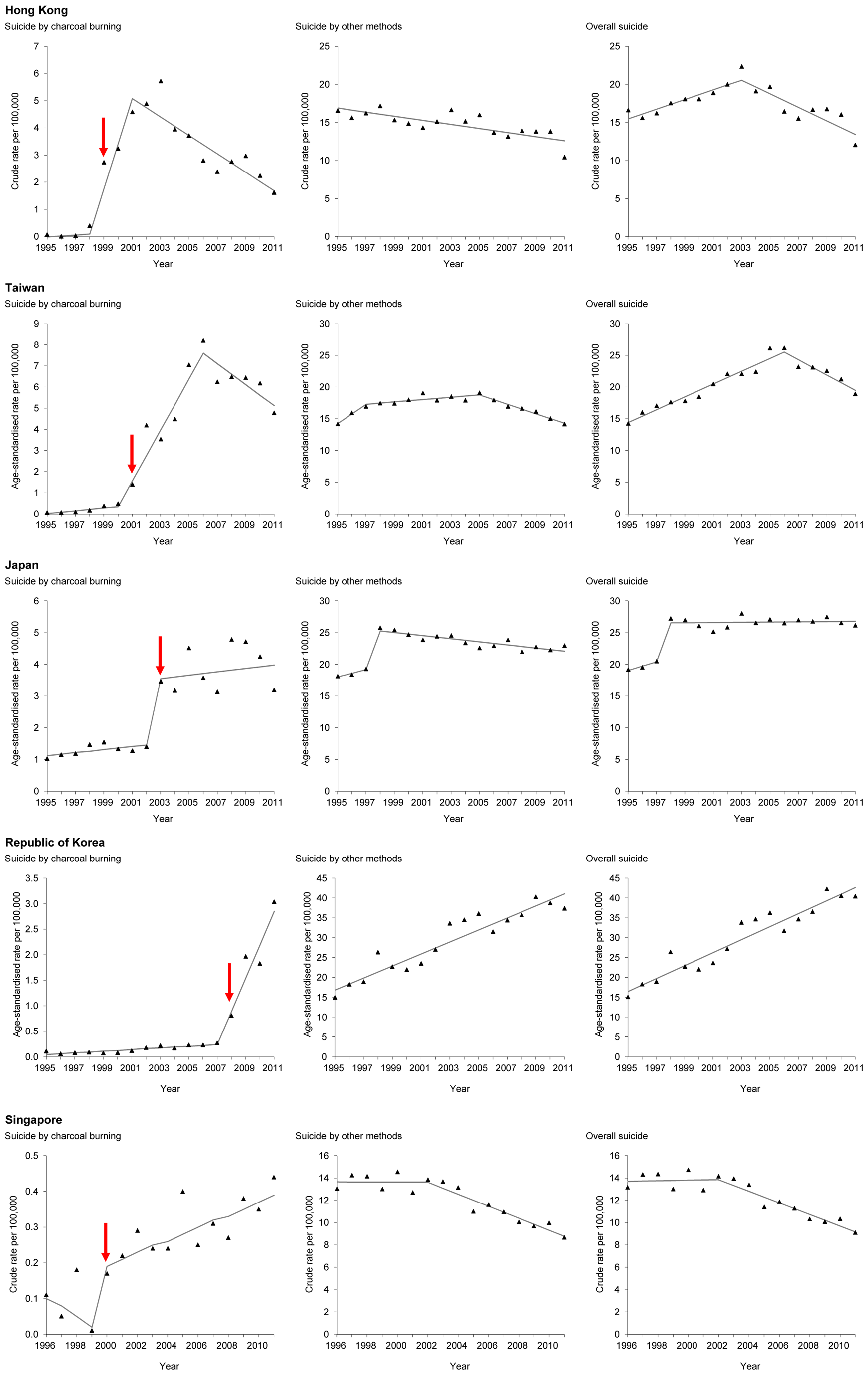

Supplement: Figure S2 — Time trends in suicide rates: charcoalburning suicide, suicide by other methods, and overall suicide, with linear trends from joinpoint regression analysis. Arrows indicate the years when charcoal-burning suicides started to increase. (TIF) [file pmed.1001622.s006.tif]

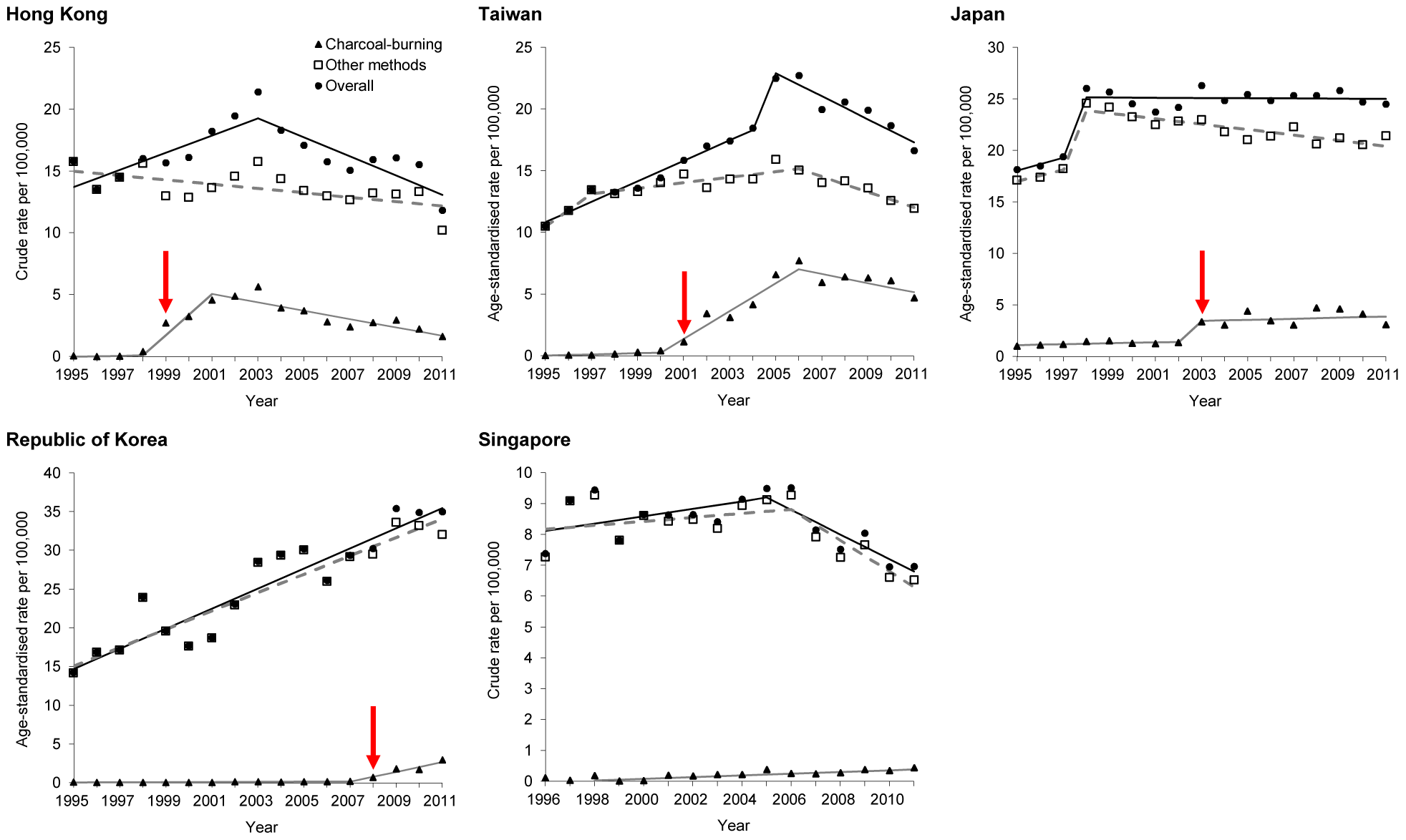

Supplement: Figure S3 — Time trends in suicide rates by method, with linear trends from joinpoint regression analysis (using certified suicide cases). Arrows indicate the years when charcoal-burning suicides started to increase. (TIF) [file pmed.1001622.s007.tif]
