# Supplementary material for: Regional Changes in Charcoal-Burning Suicide Rates in East/Southeast Asia from 1995 to 2011: A Time Trend Analysis
Source: PLoS Med. 2014 Apr 1;11(4):e1001622. doi: 10.1371/journal.pmed.1001622 (PMC3972087; doi:10.1371/journal.pmed.1001622)
Supplement: Table S1 — Number, percent, and rate per 100,000 of charcoal-burning suicide (based on certified suicide cases only). (DOC) [file pmed.1001622.s008.doc]

### Table S1. Number, percent and rate^a^ per 100,000 of charcoal-burning suicide (based on certified suicide only).

|  | Hong Kong | | |  | Taiwan | | |  | Japan^b^ | | |  | South Korea | | |  | Singapore | | |
| --- | --- | --- | --- | --- | --- | --- | --- | --- | --- | --- | --- | --- | --- | --- | --- | --- | --- | --- | --- |
| Year | Number | (%) | Rate |  | Number | (%) | Rate |  | Number | (%) | Rate |  | Number | (%) | Rate |  | Number | (%) | Rate |
| **Males and females** | | | | | | | | | | | | | | | | | | | |
| 1995 | 3 | (0.4) | 0.1 |  | 6 | (0.4) | 0.0 |  | 1069 | (5.0) | 1.0 |  | 36 | (0.8) | 0.1 |  |  |  |  |
| 1996 | 0 | (0.0) | 0.0 |  | 7 | (0.4) | 0.0 |  | 1187 | (5.4) | 1.1 |  | 19 | (0.3) | 0.1 |  | 4 | (1.5) | 0.1 |
| 1997 | 1 | (0.1) | 0.0 |  | 10 | (0.5) | 0.1 |  | 1254 | (5.4) | 1.2 |  | 22 | (0.4) | 0.1 |  | 1 | (0.3) | 0.0 |
| 1998 | 21 | (2.4) | 0.3 |  | 25 | (1.2) | 0.1 |  | 1590 | (5.1) | 1.4 |  | 28 | (0.3) | 0.1 |  | 7 | (1.9) | 0.2 |
| 1999 | 147 | (17.2) | 2.5 |  | 48 | (2.1) | 0.3 |  | 1648 | (5.3) | 1.5 |  | 16 | (0.2) | 0.0 |  | 0 | (0.0) | 0.0 |
| 2000 | 179 | (20.1) | 3.1 |  | 72 | (2.9) | 0.4 |  | 1424 | (4.8) | 1.3 |  | 18 | (0.3) | 0.0 |  | 1 | (0.3) | 0.0 |
| 2001 | 256 | (25.0) | 4.3 |  | 207 | (7.5) | 1.1 |  | 1365 | (4.7) | 1.2 |  | 25 | (0.4) | 0.1 |  | 8 | (2.2) | 0.2 |
| 2002 | 277 | (25.1) | 4.7 |  | 627 | (20.6) | 3.4 |  | 1510 | (5.1) | 1.4 |  | 53 | (0.6) | 0.1 |  | 7 | (1.9) | 0.2 |
| 2003 | 320 | (26.3) | 5.4 |  | 576 | (18.1) | 3.1 |  | 3536 | (11.1) | 3.4 |  | 65 | (0.6) | 0.2 |  | 9 | (2.6) | 0.2 |
| 2004 | 227 | (21.5) | 3.8 |  | 779 | (22.5) | 4.1 |  | 3189 | (10.6) | 3.1 |  | 50 | (0.4) | 0.1 |  | 9 | (2.4) | 0.2 |
| 2005 | 215 | (21.5) | 3.4 |  | 1251 | (29.3) | 6.6 |  | 4483 | (14.8) | 4.4 |  | 62 | (0.5) | 0.2 |  | 16 | (4.0) | 0.4 |
| 2006 | 165 | (17.7) | 2.7 |  | 1477 | (33.6) | 7.7 |  | 3462 | (11.7) | 3.5 |  | 64 | (0.6) | 0.2 |  | 11 | (2.6) | 0.2 |
| 2007 | 143 | (15.8) | 2.3 |  | 1152 | (29.4) | 5.9 |  | 3044 | (9.9) | 3.1 |  | 84 | (0.7) | 0.2 |  | 11 | (2.9) | 0.2 |
| 2008 | 166 | (17.2) | 2.7 |  | 1254 | (30.5) | 6.4 |  | 4347 | (14.5) | 4.7 |  | 292 | (2.3) | 0.7 |  | 13 | (3.6) | 0.3 |
| 2009 | 180 | (18.3) | 2.8 |  | 1249 | (30.9) | 6.3 |  | 4341 | (14.3) | 4.6 |  | 765 | (5.0) | 1.8 |  | 19 | (4.7) | 0.4 |
| 2010 | 137 | (14.3) | 2.1 |  | 1209 | (31.2) | 6.1 |  | 3914 | (13.3) | 4.1 |  | 727 | (4.7) | 1.7 |  | 18 | (5.1) | 0.4 |
| 2011 | 101 | (13.7) | 1.5 |  | 938 | (26.8) | 4.7 |  | 2880 | (10.0) | 3.1 |  | 1250 | (7.9) | 3.0 |  | 23 | (6.4) | 0.4 |
| **Males** | | | | | | | | | | | | | | | | | | | |
| 1995 | 3 | (0.6) | 0.1 |  | 4 | (0.4) | 0.0 |  | 945 | (6.7) | 1.8 |  | 23 | (0.7) | 0.1 |  |  |  |  |
| 1996 | 0 | (0.0) | 0.0 |  | 6 | (0.5) | 0.1 |  | 1029 | (7.0) | 1.9 |  | 16 | (0.4) | 0.1 |  |  |  |  |
| 1997 | 1 | (0.2) | 0.0 |  | 8 | (0.6) | 0.1 |  | 1093 | (7.0) | 2.0 |  | 17 | (0.4) | 0.1 |  |  |  |  |
| 1998 | 14 | (2.6) | 0.5 |  | 24 | (1.7) | 0.3 |  | 1410 | (6.4) | 2.6 |  | 22 | (0.4) | 0.1 |  |  |  |  |
| 1999 | 109 | (20.1) | 3.8 |  | 47 | (3.0) | 0.5 |  | 1470 | (6.7) | 2.7 |  | 12 | (0.2) | 0.1 |  |  |  |  |
| 2000 | 116 | (21.4) | 4.2 |  | 65 | (4.0) | 0.7 |  | 1288 | (6.0) | 2.3 |  | 14 | (0.3) | 0.1 |  |  |  |  |
| 2001 | 166 | (25.8) | 5.9 |  | 159 | (8.4) | 1.7 |  | 1225 | (5.9) | 2.2 |  | 21 | (0.4) | 0.1 |  |  |  |  |
| 2002 | 195 | (26.4) | 6.8 |  | 466 | (22.1) | 5.0 |  | 1353 | (6.3) | 2.4 |  | 47 | (0.8) | 0.2 |  |  |  |  |
| 2003 | 234 | (29.2) | 8.3 |  | 423 | (19.7) | 4.5 |  | 3120 | (13.5) | 5.9 |  | 53 | (0.7) | 0.3 |  |  |  |  |
| 2004 | 164 | (24.6) | 5.9 |  | 562 | (24.0) | 5.9 |  | 2778 | (12.8) | 5.3 |  | 41 | (0.5) | 0.2 |  |  |  |  |
| 2005 | 146 | (23.8) | 4.9 |  | 915 | (30.8) | 9.5 |  | 3879 | (17.6) | 7.5 |  | 46 | (0.6) | 0.2 |  |  |  |  |
| 2006 | 112 | (19.5) | 3.9 |  | 1112 | (36.1) | 11.5 |  | 2977 | (14.0) | 5.9 |  | 53 | (0.7) | 0.3 |  |  |  |  |
| 2007 | 96 | (16.5) | 3.3 |  | 830 | (31.5) | 8.5 |  | 2614 | (12.0) | 5.2 |  | 65 | (0.8) | 0.3 |  |  |  |  |
| 2008 | 107 | (18.8) | 3.9 |  | 904 | (32.1) | 9.2 |  | 3579 | (16.8) | 7.5 |  | 227 | (2.8) | 1.1 |  |  |  |  |
| 2009 | 120 | (19.5) | 4.1 |  | 910 | (32.7) | 9.1 |  | 3626 | (16.5) | 7.5 |  | 628 | (6.4) | 2.9 |  |  |  |  |
| 2010 | 92 | (15.4) | 3.1 |  | 881 | (33.5) | 8.8 |  | 3271 | (15.7) | 6.8 |  | 608 | (5.9) | 2.8 |  |  |  |  |
| 2011 | 76 | (16.8) | 2.5 |  | 681 | (28.6) | 6.8 |  | 2406 | (12.2) | 5.1 |  | 1046 | (9.7) | 4.8 |  |  |  |  |
| **Females** | | | | | | | | | | | | | | | | | | | |
| 1995 | 0 | (0.0) | 0.0 |  | 2 | (0.4) | 0.0 |  | 124 | (1.7) | 0.2 |  | 13 | (0.9) | 0.1 |  |  |  |  |
| 1996 | 0 | (0.0) | 0.0 |  | 1 | (0.2) | 0.0 |  | 158 | (2.2) | 0.3 |  | 3 | (0.2) | 0.0 |  |  |  |  |
| 1997 | 0 | (0.0) | 0.0 |  | 2 | (0.3) | 0.0 |  | 161 | (2.1) | 0.3 |  | 5 | (0.3) | 0.0 |  |  |  |  |
| 1998 | 7 | (2.2) | 0.2 |  | 1 | (0.1) | 0.0 |  | 180 | (1.9) | 0.3 |  | 6 | (0.3) | 0.0 |  |  |  |  |
| 1999 | 38 | (12.1) | 1.3 |  | 1 | (0.1) | 0.0 |  | 178 | (2.0) | 0.3 |  | 4 | (0.2) | 0.0 |  |  |  |  |
| 2000 | 63 | (18.1) | 2.1 |  | 7 | (0.9) | 0.1 |  | 136 | (1.6) | 0.3 |  | 4 | (0.2) | 0.0 |  |  |  |  |
| 2001 | 90 | (23.7) | 2.8 |  | 48 | (5.5) | 0.5 |  | 140 | (1.7) | 0.3 |  | 4 | (0.2) | 0.0 |  |  |  |  |
| 2002 | 82 | (22.5) | 2.7 |  | 161 | (17.2) | 1.8 |  | 157 | (1.9) | 0.3 |  | 6 | (0.2) | 0.0 |  |  |  |  |
| 2003 | 86 | (20.7) | 2.7 |  | 153 | (14.8) | 1.7 |  | 416 | (4.8) | 0.8 |  | 12 | (0.4) | 0.1 |  |  |  |  |
| 2004 | 63 | (16.1) | 2.0 |  | 217 | (19.5) | 2.3 |  | 411 | (5.0) | 0.8 |  | 9 | (0.3) | 0.0 |  |  |  |  |
| 2005 | 69 | (17.9) | 2.1 |  | 336 | (25.8) | 3.6 |  | 604 | (7.3) | 1.2 |  | 16 | (0.4) | 0.1 |  |  |  |  |
| 2006 | 53 | (14.8) | 1.6 |  | 365 | (27.8) | 3.8 |  | 485 | (5.7) | 1.0 |  | 11 | (0.3) | 0.1 |  |  |  |  |
| 2007 | 47 | (14.6) | 1.4 |  | 322 | (25.2) | 3.4 |  | 430 | (4.9) | 0.9 |  | 19 | (0.4) | 0.1 |  |  |  |  |
| 2008 | 59 | (14.8) | 1.8 |  | 350 | (26.9) | 3.6 |  | 768 | (8.9) | 1.8 |  | 65 | (1.4) | 0.3 |  |  |  |  |
| 2009 | 60 | (16.3) | 1.7 |  | 339 | (26.9) | 3.6 |  | 715 | (8.4) | 1.6 |  | 137 | (2.5) | 0.7 |  |  |  |  |
| 2010 | 45 | (12.5) | 1.3 |  | 328 | (26.3) | 3.3 |  | 643 | (7.6) | 1.4 |  | 119 | (2.3) | 0.6 |  |  |  |  |
| 2011 | 25 | (8.7) | 0.7 |  | 257 | (23.1) | 2.6 |  | 474 | (5.3) | 1.1 |  | 204 | (4.1) | 1.0 |  |  |  |  |

^a^ Age-standardised rate except Singapore, for which rates were crude rates.

^b^ In Japan figures for 2008 onwards included not only charcoal-burning suicides but also some deaths from hydrogen sulphide poisoning, which increased markedly in 2008 [31].
