# Supplementary material for: Regional Changes in Charcoal-Burning Suicide Rates in East/Southeast Asia from 1995 to 2011: A Time Trend Analysis
Source: PLoS Med. 2014 Apr 1;11(4):e1001622. doi: 10.1371/journal.pmed.1001622 (PMC3972087; doi:10.1371/journal.pmed.1001622)
Supplement: Table S2 — Summary of the mean annual increases in suicide rate (based on certified suicide cases only) per 100,000 and join points for time trends in five East/ Southeast Asian countries, 1995–2011. (DOC) [file pmed.1001622.s009.doc]

### Table S2. Summary of the mean annual increases in suicide rates^a^ (based on certified suicide only) per 100,000 (β) and join points (JPs) for time trends in five East/Southeast Asian countries, 1995-2011^b^.

|  |  | Segment 1^c^ | |  |  | Segment 2^c^ | |  |  | Segment 3^c^ | |
| --- | --- | --- | --- | --- | --- | --- | --- | --- | --- | --- | --- |
| Country |  | β | (95% CI) | JP 1^c^ | (95% CI) | β | (95% CI) | JP 2^c^ | (95% CI) | β | (95% CI) |
| Hong Kong | Charcoal-burning suicide | 0.03 | (-0.06, 0.12) | 1998 | (1997, 1999) | 1.66 | (0.22, 3.09) | 2001 | (1999, 2004) | -0.34 | (-0.45, -0.23) |
|  | Suicide by other methods | -0.18 | (-0.29, -0.07) |  |  |  |  |  |  |  |  |
|  | Overall suicide | 0.69 | (0.30, 1.08) | 2003 | (2001, 2005) | -0.77 | (-1.12, -0.42) |  |  |  |  |
|  |  |  |  |  |  |  |  |  |  |  |  |
| Taiwan | Charcoal-burning suicide | 0.05 | (-0.01, 0.11) | 2000 | (1999, 2001) | 1.13 | (0.81, 1.45) | 2006 | (2004, 2008) | -0.38 | (-0.80, 0.05) |
|  | Suicide by other methods | 1.33 | (-0.06, 2.73) | 1997 | (1997, 2002) | 0.22 | (0.06, 0.38) | 2006 | (2004, 2009) | -0.62 | (-0.91, -0.33) |
|  | Overall suicide | 0.83 | (0.67, 0.99) | 2004 | (1999, 2005) | 4.61 | -^d^ | 2005 | (2004, 2007) | -0.93 | (-1.25, -0.62) |
|  |  |  |  |  |  |  |  |  |  |  |  |
| Japan | Charcoal-burning suicide | 0.04 | (-0.07, 0.16) | 2002 | (1999, 2003) | 2.04 | -^d^ | 2003 | (2002, 2009) | 0.05 | (-0.12, 0.23) |
|  | Suicide by other methods | 0.55 | (-0.92, 2.01) | 1997 | (1997, 1998) | 5.77 | -^d^ | 1998 | (1998, 1999) | -0.27 | (-0.36, -0.18) |
|  | Overall suicide | 0.63 | (-1.11, 2.36) | 1997 | (1997, 1998) | 5.83 | -^d^ | 1998 | (1998, 1999) | -0.01 | (-0.12, 0.10) |
|  |  |  |  |  |  |  |  |  |  |  |  |
| South Korea | Charcoal-burning suicide | 0.01 | (0.00, 0.02) | 2007 | (2006, 2008) | 0.64 | (0.47, 0.81) |  |  |  |  |
|  | Suicide by other methods | 1.19 | (0.94, 1.43) |  |  |  |  |  |  |  |  |
|  | Overall suicide | 0.64 | (0.39, 0.89) |  |  |  |  |  |  |  |  |
|  |  |  |  |  |  |  |  |  |  |  |  |
| Singapore | Charcoal-burning suicide | 0.03 | (0.02, 0.04) |  |  |  |  |  |  |  |  |
|  | Suicide by other methods | 0.06 | (-0.06, 0.19) | 2006 | (2003, 2008) | -0.50 | (-0.80, -0.21) |  |  |  |  |
|  | Overall suicide | 0.12 | (-0.04, 0.28) | 2005 | (2002, 2008) | -0.40 | (-0.66, -0.14) |  |  |  |  |
| ^a^ Age-standardised rates for Taiwan, Japan, and South Korea; crude rates for Hong Kong and Singapore. | | | | | | | | |  |  |  |
| ^b^ Except Singapore (1996-2011). | |  |  |  |  |  |  |  |  |  |  |
| ^c^ Segments were linear trends between join points (JPs, i.e. the years when the trends changed) identified using joinpoint regression, which characterises time trends as contiguous linear segments and join points.. | | | | | | | | | | | |
| ^d^ 95% CI could not be estimated by the joinpoint regression as the segment included only two data points. | | | | | | | | |  |  |  |

### 
