# Supplementary material for: Regional Changes in Charcoal-Burning Suicide Rates in East/Southeast Asia from 1995 to 2011: A Time Trend Analysis
Source: PLoS Med. 2014 Apr 1;11(4):e1001622. doi: 10.1371/journal.pmed.1001622 (PMC3972087; doi:10.1371/journal.pmed.1001622)
Supplement: Table S3 — Summary of the mean annual increases in suicide rate per 100,000 and join points for time trends, accounting for autocorrelation, in five East/Southeast Asian countries, 1995–2011. (DOC) [file pmed.1001622.s010.doc]

### Table S3. Summary of the mean annual increases in suicide rates^a^ per 100,000 (β) and join points (JPs) for time trends, accounting for autocorrelation, in five East/Southeast Asian countries, 1995-2011^b^.

|  |  | Segment 1^c^ | |  |  | Segment 2^c^ | |  |  | Segment 3^c^ | |
| --- | --- | --- | --- | --- | --- | --- | --- | --- | --- | --- | --- |
| Country |  | β | (95% CI) | JP 1^c^ | (95% CI) | β | (95% CI) | JP 2^c^ | (95% CI) | β | (95% CI) |
| Hong Kong | Charcoal-burning suicide | 0.04 | (-0.11, 0.19) | 1998 | (1997, 1999) | 1.66 | (0.14, 3.19) | 2001 | (1999, 2004) | -0.34 | (-0.45, -0.23) |
|  | Suicide by other methods | -0.25 | (-0.35, -0.16) |  |  |  |  |  |  |  |  |
|  | Overall suicide | 0.63 | (0.33, 0.94) | 2003 | (2001, 2005) | -0.85 | (-1.13, -0.57) |  |  |  |  |
|  |  |  |  |  |  |  |  |  |  |  |  |
| Taiwan | Charcoal-burning suicide | 0.07 | (0.00, 0.14) | 2000 | (1999, 2001) | 1.21 | (0.90, 1.52) | 2006 | (2004, 2007) | -0.49 | (-0.89, -0.08) |
|  | Suicide by other methods | 1.53 | (-0.05, 3.11) | 1997 | (1997, 2002) | 0.18 | (0.05, 0.31) | 2005 | (2003, 2007) | -0.73 | (-0.89, -0.58) |
|  | Overall suicide | 1.01 | (0.86, 1.15) | 2006 | (2005, 2007) | -1.20 | (-1.68, -0.72) |  |  |  |  |
|  |  |  |  |  |  |  |  |  |  |  |  |
| Japan | Charcoal-burning suicide | 0.05 | (-0.05, 0.14) | 2002 | (2000, 2003) | 2.08 | -^d^ | 2003 | (2002, 2009) | 0.07 | (-0.08, 0.22) |
|  | Suicide by other methods | 0.64 | (-1.02, 2.30) | 1997 | (1997, 1998) | 5.99 | -^d^ | 1998 | (1998, 1999) | -0.25 | (-0.32, -0.17) |
|  | Overall suicide | 0.66 | (-1.08, 2.41) | 1997 | (1997, 1998) | 6.13 | -^d^ | 1998 | (1998, 1999) | 0.02 | (-0.09, 0.13) |
|  |  |  |  |  |  |  |  |  |  |  |  |
| South Korea | Charcoal-burning suicide | 0.02 | (0.01, 0.02) | 2007 | (2006, 2008) | 0.65 | (0.54, 0.76) |  |  |  |  |
|  | Suicide by other methods | 1.52 | (1.28, 1.75) |  |  |  |  |  |  |  |  |
|  | Overall suicide | 0.65 | (0.41, 0.89) |  |  |  |  |  |  |  |  |
|  |  |  |  |  |  |  |  |  |  |  |  |
| Singapore | Charcoal-burning suicide | -0.03 | (-0.08, 0.02) | 1999 | (1998, 2001) | 0.11 | -^d^ | 2001 | (1999, 2003) | 0.01 | (0.01, 0.02) |
|  | Suicide by other methods | -0.03 | (-0.27, 0.20) | 2002 | (1999, 2004) | -0.53 | (-0.63, -0.43) |  |  |  |  |
|  | Overall suicide | 0.00 | (-0.23, 0.23) | 2002 | (2000, 2004) | -0.51 | (-0.61, -0.41) |  |  |  |  |
| ^a^ Age-standardised rates for Taiwan, Japan, and South Korea; crude rates for Hong Kong and Singapore. | | | | | | | | |  |  |  |
| ^b^ Except Singapore (1996-2011). | |  |  |  |  |  |  |  |  |  |  |
| ^c^ Segments were linear trends between join points (JPs, i.e. the years when the trends changed) identified using joinpoint regression, which characterises time trends as contiguous linear segments and join points.. | | | | | | | | | | | |
| ^d^ 95% CI could not be estimated by the joinpoint regression as the segment included only two data points. | | | | | | | | |  |  |  |
